# Combinatorial Library of Improved Peptide Aptamers, CLIPs to Inhibit RAGE Signal Transduction in Mammalian Cells

**DOI:** 10.1371/journal.pone.0065180

**Published:** 2013-06-13

**Authors:** Sergey Reverdatto, Vivek Rai, Jing Xue, David S. Burz, Ann Marie Schmidt, Alexander Shekhtman

**Affiliations:** 1 Department of Chemistry, State University of New York at Albany, Albany, New York, United States of America; 2 Langone Medical Center, New York University, New York, New York, United States of America; Bioinformatics Institute, Singapore

## Abstract

Peptide aptamers are small proteins containing a randomized peptide sequence embedded into a stable protein scaffold, such as Thioredoxin. We developed a robust method for building a **C**ombinatorial **L**ibrary of **I**mproved **P**eptide aptamer**s** (CLIPs) of high complexity, containing ≥3×10^10^ independent clones, to be used as a molecular tool in the study of biological pathways. The Thioredoxin scaffold was modified to increase solubility and eliminate aggregation of the peptide aptamers. The CLIPs was used in a yeast two-hybrid screen to identify peptide aptamers that bind to various domains of the Receptor for Advanced Glycation End products (RAGE). NMR spectroscopy was used to identify interaction surfaces between the peptide aptamers and RAGE domains. Cellular functional assays revealed that in addition to directly interfering with known binding sites, peptide aptamer binding distal to ligand sites also inhibits RAGE ligand-induced signal transduction. This finding underscores the potential of using CLIPs to select allosteric inhibitors of biological targets.

## Introduction

Designing molecules that modulate cellular processes through selective high affinity binding to discreet sites on biological molecules is a “Holy Grail” of bioengineering [1]. “Designer” nucleic acids and proteins have been used successfully for this purpose due to the ease in generating large combinatorial libraries [2]–[5], up to 10^13^ unique molecules. Nucleic acid-based tools (DNA and RNA aptamers) are very popular due to the ease of identifying high affinity binders for selected interactions [6]. Nonetheless, peptide-derived probes provide a distinct advantage as biochemical research instruments: Peptide aptamers generally exhibit a smaller binding footprint allowing for a more thorough and precise interrogation of the target than that afforded by nucleic acid-based probes [7].

There are both *in vivo* and *in vitro* approaches to select peptide aptamers for a particular target. The most commonly used *in vitro* methods, phage [8], ribosome [9], and mRNA [4] display, utilize several rounds of peptide enrichment through *in vitro* binding to the protein target. Selected binders stay attached to the phage particle, ribosome, or mRNA molecule correspondingly, permitting the recovery of sequence information. *In vitro* methods allow the construction of very large combinatorial libraries, up to 10^15^ unique molecules in the case of mRNA display [4], and rapid screening protocols, but may suffer from bias introduced by the presence of peptide inserts incompatible with virion assembly, secretion or infection [10], binding to components of the screening system other than the target molecule, generating Target Unrelated Peptides (TUPs) [11], [12], or by developing phage clones with propagation advantages that can severely affect library diversity [13]–[15]. *In vitro* selection usually requires a substantial amount of purified target, which is not always readily available, and selection is carried out outside a cellular environment, which may lead to improper folding or the lack of required post-translational modifications. Importantly, competition between many potential ligands for a limited number of binding sites on the target can result in the failure to identify potential interactors [16].

Although time consuming [17], *in vivo* selection, such as the Yeast two Hybrid (Y2H) and similar techniques, is preferable to *in vitro* screening because the selection occurs under near physiological conditions and does not require purified targets. An important feature that distinguishes Y2H screening is that each peptide aptamer expressed within a single cell has no competition for binding to a given target during selection [16]. This makes possible isolation of peptide aptamers with different affinities that bind to distinct or even overlapping sites on the same target. The resulting collection of isolated peptide aptamers allows for the comprehensive characterization of interaction surfaces on target molecules *in vitro* and *in vivo*.

The concept of peptide aptamers, originally introduced by Roger Brent [18], envisaged a short amino acid sequence embedded or constrained within a small and very stable protein backbone or scaffold. Conformational constraining, which stabilizes the insert loop and makes it more likely to fold and recognize cognate surfaces, appears to be critical for high affinity binding, since in at least one case a constrained aptamer exhibited a 1000-fold greater affinity compared to the unconstrained peptide [19]. Bacterial Thioredoxin A (TrxA) is among the most widely used scaffold for *in vivo* selection; it is a well characterized, small, rigid, rapidly folding protein, which possesses superior stability over other scaffolds [20]; making it an ideal choice for use in constructing a peptide aptamer library.

The first generations of combinatorial libraries for in vivo screening utilized non-directional cloning at a singular Rsr II restriction site [18], which resulted in a limited library size, up to 10^8^ unique molecules, and decreased the chances of obtaining successful target hits. Another limitation was the difficulty in translating library diversity, contained in ligation reactions, into corresponding numbers of colonies on selection plates, which is a function of transformation efficiency [21]. Finally, random peptide sequence insertions frequently destabilized the Thioredoxin scaffold and might create molecules that are prone to aggregation [22], [23]. In this work, we successfully addressed these problems and developed a robust method for constructing a Combinatorial Library of Improved Peptide aptamers (CLIPs).

Using the Receptor for Advanced Glycated End products (RAGE) [24] as a model system in a Y2H screen, we isolated several peptide aptamers that bind to distinct sites on RAGE with high affinity and affect the RAGE-dependent signal transduction cascade induced by ligand binding. Signal transduction of RAGE is implicated in the etiology of many diseases, including diabetes, neurodegeneracy, cancer, and inflammation [25]–[28]. The binding of RAGE ligands, such as S100B protein [27], leads to structural rearrangement of the receptor and results in phosphorylation of RAGE effectors [29]–[31].

Until now the broad application of peptide aptamers as molecular tools to study complex biological pathways and as drug candidates has been hampered by the difficulty in constructing large libraries expressing soluble products. The removal of those limitations will pave the way for structural characterization of peptide aptamers interacting with targets by high resolution NMR spectroscopy or X-ray crystallography and, subsequently, the use of peptide aptamers for interrogating, visualizing and modulating key cellular processes [32].

## Materials and Methods

### Reagents and Chemicals

All enzymes were acquired from New England Biolabs. Anti-HA and anti-Myc antibodies were purchased from Rockland (Gilbertsville, PA), all other antibodies were from Cell Signaling Technology (Danvers, MA), unless otherwise specified. All other chemicals used were reagent grade or better.

### Cloning, library construction

All routine cloning was performed using *E.coli* strain DH10B. Plasmid pET32 (Novagen) was used as a source of Thioredoxin DNA. To facilitate directional PA insertion, the original bacterial TrxA scaffold (TSc) was modified. Nucleotides 96–110 in the Thioredoxin sequence, which contain two Cys codons and an Rsr II site was replaced by a BamHI-EcoRI-ApaI polylinker by using mutating oligonucleotides (**Table S1**) and the Stratagene QuikChange II XL kit. Yeast shuttle plasmid pGADT7 (Clontech) was used to construct the PA library vector, pLib2. The polylinker region between the NdeI and XhoI sites of pGADT7 was replaced with short sequence containing a diagnostic XbaI site and a stop codon in-frame with the GAL4 activation domain (AD)/HA epitope fusion (**Table S1**). The modified TSc was cloned in-frame at the KpnI site (bp 1552) located between the SV40 Nuclear Localization Signal (NLS) and GAL4 activation domain sequence. pLib2 constitutively expresses a NLS/modified-TrxA/GAL4AD/HA fusion protein from the ADH promoter, and contains LEU2 (yeast) and Amp (bacteria) selection markers.

A set of probes for interrogating the Y2H library was generated by inserting fragments of human RAGE cDNA into yeast vector pGBKT7 (Clontech) between the NdeI and SalI restriction sites in frame with the GAL4 binding domain (BD)/c-Myc epitope fusion. Fragments corresponding to amino acids 23–125 (V domain), 103–247 (extended C1 domain), 231–334 (C2 domain) and 24–334 (sRAGE) of full-length RAGE protein were PCR-amplified from human RAGE cDNA library clone BC020669 (Open Biosystems) using oligonucleotides primers designed to incorporate the appropriate flanking sites (**Table S1**). The resulting plasmids constitutively express GAL4BD/c-Myc/RAGE domain fusion proteins from the ADH promoter and contain TRP1 and Kan markers for selection in yeast and bacteria correspondingly.

Construction of plasmids for bacterial expression, pET28C2 and pET15b-VC1, containing human RAGE C2 domain (amino acids 235–336) and VC1 domains (amino acids 23–243), was described earlier [31],[33]. For ELISA experiments, probes consisting of RAGE fragments fused to GFP protein were prepared by cloning the human RAGE VC1 domain (amino acids 23–246 and C2 domain (amino acids 231–336) into expression vector pRSET/EmGFP (Invitrogen). Corresponding DNA fragments were PCR-amplified from human RAGE cDNA library clone BC020669 (Open Biosystems) using oligonucleotide primers (**Table S1**) designed to incorporate flanking 5′-ClaI and 3′-EcoRI restriction sites. Digested PCR products were ligated in-frame into the matching sites of the vector upstream of the GFP sequence to provide pRSET/RAGEvc1-EmGFP and pRSET/RAGEc2-EmGFP constructs. The resulting plasmids confer ampicillin resistance and express RAGE domain/GFP fusion proteins from an inducible T7 promoter.

### Y2H library screening

The commercially available Matchmaker™ Gold Yeast Two-Hybrid kit (Clontech) was used to perform the library screening. RAGE-domain bait constructs were transformed into the yeast host, Y2Hgold (MATa, trp1-901, leu2-3, 112, ura3-52, his3-200, gal4Δ, gal80Δ, LYS2::GAL1_UAS_–Gal1_TATA_–His3, GAL2_UAS_–Gal2_TATA_–Ade2, URA3::MEL1_UAS_–Mel1_TATA_, AUR1-C MEL1). The CLIPs prey was transformed into yeast strain pJ69-4α (MATα, trp1-901, leu2-3, 112, ura3-52, his3-200, gal4Δ, gal80Δ, LYS2::GAL1-HIS3, GAL2-ADE2, met2::GAL7-lacZ) [34], which has 4–5 times higher transformation efficiency compared to the Y187 strain supplied with the kit. Out of several transformation protocols tested, the one provided by Clontech yielded the highest transformation efficiencies (∼10^6^ cfu/μg), and was used for all experiments.

A sample subset of CLIPs prey containing 1.5×10^7^ independent clones was selected for screening. 1 mg of plasmid DNA was introduced into yeast strain pJ69-4α generating a pool of 10^9^ Leu^+^ yeast cells. A 30-fold increase in amount of CLIPs-containing cells was achieved by growing the library in Leu^−^ medium for 2 days [17],after which cells were aliquoted and frozen. The freezing efficiency of the library stock, determined as the ratio of the number of viable Leu^+^ yeast cells obtained upon thawing to the amount of input cells, varied between 10–50%.

### Size exclusion chromatography

Purification of Ligation products were purified by using a Sephacryl S-500HR column (GE Healthcare) attached to a BioLogic DuoFlow Chromatography system (BioRad). Separation was run under isocratic conditions, in buffer consisting of 10 mM Tris-HCl, pH 8.0, 150 mM NaCl and 1 mM EDTA. Flow rates varied between 0.1 and 0.5 mL/min.

### Expression, labeling, and purification of peptide aptamers

To purify selected peptide aptamers, the corresponding DNA fragments, obtained by yeast colony PCR, were re-amplified by using oligonucleotide primers (**Table S1**) to add 5′ Nco I and 3′ Hind III restriction sites along with a 5′-terminal His tag (**Figure S1**). Aptamers were cloned into the L-arabinose-inducible vector pBAD (Invitrogen), and introduced into *E.coli* strains DH10B or Origami.

Unlabeled PAs were routinely expressed in auto-induction medium ZYM-505 [35] supplemented with 0.05% L-arabinose and 150 μg/L of carbenicillin. Cultures were grown for 20–26 hours at 37°C, maintaining at least a 1∶10 culture-to-flask volume. To prepare uniformly labeled [*U-* ^15^N] proteins, PA constructs were transformed into *E. coli* strain Origami B and over-expressed in N-505 auto-induction medium [35] containing 2.66 g/L of ^15^N-ammonium chloride as the sole nitrogen source, 0.05% L-arabinose and 150 μg/L of carbenicillin. For [*U-* ^13^C,^15^N] labeling, cells were grown in C-505 medium [35] containing 150 μg/L of carbenicillin, 0.05% L-arabinose, 2.66 g/L ^15^N-ammonium chloride, and 7.5 mL/L of [*U-* ^13^C] glycerol as the sole carbon source. In all instances cells were grown in a shaker at 37°C, 300–350 rpm for 20–26 hours. For some labeling experiments standard minimal medium (M9) was used as well [36].

Though PAs can be purified under both non-denaturing and denaturing conditions, we found that PAs purified under denaturing conditions consistently result in higher purity of the final sample. Cells were harvested, re-suspended in extraction buffer (50 mM sodium phosphate, pH 7.0, 300 mM Sodium chloride, 6 M guanidine hydrochloride,) and sonicated. The lysate was centrifuged at 40000 ***g*** for an hour, and the supernatant was loaded onto a pre-equilibrated TALON column (cobalt affinity resin, Clontech). The column was washed with 10 column volumes (CV) of extraction buffer, 10 CV of extraction buffer containing 0.4% octyl phenol ethoxylate (Triton X-100, Baker), and again with 10 CV of extraction buffer. Protein was eluted with 10 CV of imidazole elution buffer (45 mM Sodium phosphate, pH 7.0, 270 mM Sodium chloride, 5.4 M Guanidine hydrochloride, 150 mM Imidazole). Fractions containing the eluted protein were concentrated to 2–2.5 mL by using an Amicon Ultra-15 centrifugal filter (Millipore) with a 3 kDa MW cutoff (MWCO), and 50 µL of 0.5 M EDTA, 6 mL of TSP buffer (20 mM Sodium phosphate, pH = 7.5, 100 mM Sodium thiosulfate) and 1 mL of glycerol were added. The protein was placed in a 1 kDa MWCO dialysis bag (Spectrum Laboratories, Inc.) and dialyzed against 1L of 50 mM Sodium phosphate, pH 8.0, 100 mM Sodium chloride, 0.5 M Guanidine hydrochloride, 2 mM EDTA, and 10% glycerol, for 5–10 hours at 4°C. The sample was further dialyzed against 2L of TSP buffer supplemented with 10% glycerol, for 10 hours at 4°C. Finally, PA was concentrated by using an Amicon Ultra-15 filter unit (3 kDa MWCO) and exchanged into TSP buffer. The final PA sample volume of 0.5–1 mL was supplemented with the manufacturers recommended amount of Complete Protease Inhibitor cocktail (Roche Applied Science) and stored at 4°C.

To prepare PAs for cell signaling experiments an extra purification step was incorporated. *E.coli* endotoxins were removed as outlined in [37], with a few changes. Briefly, after TALON column purification, imidazole was removed from the eluate and the protein was exchanged into Ni-NTA denaturing lysis buffer (100 mM Sodium phosphate, pH 8.0, 10 mM Tris-HCl, 6 M Guanidine hydrochloride) by using an Amicon Ultra-15 centrifugal filter (3 kDa MWCO). Special precautions were taken to use endotoxin-free plastic, reagents and water. All operations were performed at 4°C or on ice. The sample was loaded onto a Ni-NTA agarose column (Qiagen) pre-equilibrated with denaturing lysis buffer. The column was washed with 10 CV of Ni-NTA denaturing wash buffer (100 mM Sodium phosphate, pH 6.3, 10 mM Tris-HCl, 8 M Urea,), and with 10 CV of the denaturing wash buffer supplemented with 0.1% Triton-X114 (Sigma-Aldrich). The column was sequentially washed with decreasing amounts of denaturing buffer and increasing amounts of native Ni-NTA wash buffer (50 mM Sodium phosphate, pH 8.0, 300 mM Sodium chloride, 20 mM Imidazole), containing 0.1% Triton-X114 to facilitate refolding of the denatured PA on the column. A step-gradient was formed by successive two-fold dilutions of denaturing wash buffer ranging from 4 M to 0.5 M Urea, each step lasting for 10 CV. The final wash was 10 CV of native wash buffer without detergent. Refolded protein was eluted with 10 CV of native Ni-NTA elution buffer native (50 mM Sodium phosphate, pH 8.0, 300 mM Sodium chloride, 250 mM Imidazole). Protein was concentrated by using an Amicon Ultra-15 centrifugal filter (3 kDa MWCO), and was buffer exchanged into PBS containing 30% glycerol. Endotoxin levels were found to be <2 EU/mL, as determined by using the LAL Pyrogent® kit (Cambrex).

### Expression, labeling and purification of RAGE domains

Labeled VC1 domain of RAGE was prepared essentially as described [33]. The protocol to prepare the C2 domain was based on the procedure developed for the V domain [33],[36]. Specifically, for [*U-* ^15^N] labeling, *E. coli* BL21(DE3) Codon+ cells (Novagen), harboring pET28/RAGE(C2) plasmid [31], were grown at 37°C in minimal medium (M9) containing 35 μg/L of kanamycin and 1 g/L of [*U-* ^15^N] ammonium chloride as the sole nitrogen source. For [*U-* ^13^C,^15^N] labeling, cells were grown at 37°C in M9 medium containing 35 μg/L of kanamycin, 1 g/L of [*U-* ^15^N] ammonium chloride, and 2 g/L of [*U-* ^13^C] glucose instead of unlabeled glucose as the sole carbon source. Cells were grown to ∼0.7 A_600_ at 37°C, induced with 0.5 mM isopropyl 1-thio-β-D-galactopyranoside (IPTG), and grown overnight. Cells were harvested and re-suspended in 50 mM Hepes buffer, pH 7.0, containing 8 M urea and heat lysed at 100°C for 10 min. The lysate was centrifuged, and the supernatant was loaded onto a Ni-NTA agarose column (Qiagen). The column was washed with 50 mM Hepes buffer, pH 7.0, containing 8 M urea, and the protein was allowed to re-nature in 50 mM Hepes buffer, pH 7.0, on the column before eluting with 50 mM Hepes, pH 7.0, containing 250 mM imidazole. Fractions containing the eluted protein were pooled and dialyzed into 10 mM potassium phosphate, pH 6.5 buffer. The C-terminal His tag of the C2 domain was cut by thrombin (Novagen) at room temperature for 1 h before ion exchange chromatography on a HiTrap Q FF column (GE Healthcare). The fractions containing the target protein were eluted with a 0–1 M NaCl gradient. Protein was concentrated to 50–500 μM by using an Amicon Ultra-15 centrifugal filter (Millipore). Purity was estimated to be ∼95% by Coomassie-stained SDS-PAGE.

### Expression and purification of (VC1)RAGE-GFP and (C2)RAGE-GFP

Expression constructs pRSET/(VC1)RAGE-EmGFP and pRSET/(C2)RAGE-EmGFP were transformed into *E. coli* Origami B DE3 (Novagen) for over-expression. Bacterial cultures were grown under the same conditions as used to prepare PAs, except that ZYM-5052 auto-induction medium supplemented with 0.2% lactose and 150 μg/L of carbenicillin was used. After 36 hours at 37°C, cells were harvested by centrifugation, re-suspended in Ni-NTA native lysis buffer (50 mM Sodium phosphate, pH 8.0, 300 mM Sodium chloride, 10 mM Imidazole,) supplemented with Complete Protease Inhibitor cocktail (Roche Applied Science), and lysed by sonication on ice. Fusion proteins contained in the supernatant were purified on Ni-NTA resin according to the Qiagen protocol for purification under native conditions. To improve the yield of (VC1)RAGE-GFP protein an alternative protocol was used. Cultures were first grown in LB medium supplemented with 0.2% glucose and 150 μg/L of carbenicillin to an A_600_ of 0.6–0.7 at 37°C. Cells were centrifuged, washed with M9 salts and re-suspended in the same volume of M9 containing 150 μg/L of carbenicillin and 0.4% glycerol as a carbon source. The culture was incubated for 1–2 hours at 37°C, then switched to 30°C and induced with 1 mM IPTG for 16 hours. Harvested cells were processed as above. After purification, proteins were concentrated by using an Amicon Ultra-15 centrifugal filter, and was buffer-exchanged into PBS supplemented with 30% glycerol. Samples were stored at −80°C.

### Site-directed mutagenesis of the Thioredoxin scaffold

The QuikChange II XL site-directed mutagenesis kit (Strategene) was used, in accordance with the manufacturer's protocol, to introduce mutations into *TrxA* for the PAs already cloned into the pBAD vector (see **Table S1** for the list of mutating oligonucleotides used). Mutated plasmids were purified using a Mini-Prep kit (Qiagen) and sequenced.

To re-clone novel PAs from yeast vectors into pBAD, while simultaneously including beneficial mutations, a simplified version of overlap extension PCR cloning [38] was adopted (**Figure S1**). Briefly, oligonucleotides D26A forward and K57E reverse (**Table S1**) were used to amplify the mutating 158 bp “megaprimer” by using the aptamer fragment obtained from a particular yeast colony (see above) as a template. Parameters used were 30 seconds at 98°C for initial denaturation, followed by cycling between 98°C (7 s) and 72°C (20 s) for 35 repetitions. Phusion polymerase (New England BioLabs) was employed for all amplifications. The PCR product was purified by using the QIAquick PCR purification kit (Qiagen) and the resulting megaprimer was used to mutagenize *TrxA* cloned into pBAD using the following parameters: 30 seconds at 98°C for initial denaturation, followed by cycling between 98°C (15 s), 66°C (1 min), and 72°C (6.5 min) for 20 repetitions. Reactions were treated with DpnI in the same buffer for 2 hours at 37°C and ethanol precipitated in the presence of 0.1 mg/mL of glycogen. The DNA was dissolved in TE buffer and electroporated into *E. coli* strain DH10B. Colony PCR was used to identify mutants containing PA inserts, which were subsequently verified by sequencing. The mutation success rate was relatively high, estimated to be more than 50% correct insertions relative to the total number of produced colonies.

### ELISA assays

To estimate the binding affinities of positiveY2H interactors *in vitro*, yeast lysates were prepared by the spheroplast method [39]. Protein concentrations were adjusted to equal values with PBS. 96-well plates (Falcon ProBind, Becton Dickinson) were first coated with bacterially expressed bait protein, RAGE VC1 or C2 domain, at 50 ng/well in 0.1 M Sodium carbonate buffer, pH 9.6, and incubated overnight at 4°C. The wells were blocked with 3% BSA (fraction V, Calbiochem) and exposed to yeast lysates normalized to a protein concentration of 1 mg/mL at 4 °C for 5 h, followed by primary (anti-HA, 2 hours) and secondary (anti-rabbit HRP-conjugate, 1 hour) antibodies. All washes between steps were performed 4–6 times with TBST buffer. Chromogenic substrate (TMB, Thermo Scientific) was added and plates were examined by using a Synergy HT scanner (BioTek Instuments) at 370 or 650 nm.

To determine the binding constants, K_d_, plate wells were first covered with 200–500 ng of purified PA in 50 µL of carbonate buffer and incubated overnight at 4°C. Wells were blocked with 1% NF dry milk (Millipore) or 1% BSA in PBST (PBS containing 0.05% Tween-20) and incubated for 2 h at room temperature. RAGE VC1-GFP or C2–GFP fusion protein, dissolved in PBST at various concentrations ranging from 0 to 2000 nM, was added to the wells and incubated for 2 h at room temperature. Anti-GFP HRP-conjugate antibody (Invitrogen), diluted 1∶1000–1∶3000 in PBST containing 0.03% NF dry milk or 0.1% BSA, was added to each well and incubated for 3 h at room temperature or overnight at 4 °C. After adding chromogenic substrate, plates were scanned as described above. All washes between steps were performed 4–6 times with TBST buffer. Statistical analyses, non-linear regression analyses and curve fitting of the data were performed by using GraphPad Prism software (GraphPad Inc). The data were fitted to the equation: *A =  A_max_*×*X/(K_d_ + X)*, where *A* and *A_max_* are the absorbance and maximum absorbance of the chromogenic substrate, respectively, *X* is a concentration of either RAGE VC1-GFP or C2-GFP, and *K_d_* is a dissociation constant. The model assumed one high affinity binding site.

### Modeling of the Thioredoxin scaffold with an aptamer loop

MODELER 9v4 [40] was used to generate a model of PA structure. The protein aptamer sequence, containing an N-terminal His_6_ tag, peptide insert and scaffold mutations, was aligned with the wild type *E.coli* Thioredoxin structure (PDB code 2TRX, A-chain). The resulting alignment was used as input for MODELER 9v4 [40]. Protein modeling included a loop refinement option. The model with lowest energy scores was further minimized by using the CHARMM force field [41]. The quality of the homology model was assessed by the Verify3D structure evaluation server [42], which produced a Verify score of 51.8 (Expected High/Low Scores being 55.57/25.01). By using PROCHECK [43], we verified that 99% of the backbone conformational angles for the residues of the simulated structure fell within the most favorable and favorable regions of the Ramachandran plot.

### NMR experiments

Protein samples of [*U-* ^13^C,^15^N] and [*U-* ^15^N] VC1 domain, C2 domain and PAs #44, #55, and #103, with concentrations ranging from 60 to 100 μM were dissolved in NMR buffer (10 mM Sodium phosphate, pH 7.5, 100 mM Sodium thiosulfate, 0.02% (w/v) NaN_3_, 90%/10%H_2_O/D_2_O). Standard double and triple resonance spectra ^1^H{^15^N}-HSQC, HN(CA)CO, HNCO, HN(CO)CA, HNCA, CBCA(CO)NH, HNCACB, 3D DIPSI-HSQC and 3D NOESY-HSQC experiments [44] were acquired at 298 K using an Avance II Bruker spectrometer operating at a ^1^H frequency of 700 MHz equipped with a single X-axis gradient cryoprobe. All spectra were processed using TOPSPIN 2.1 software (Bruker, Inc). Chemical shift assignments, based on the published assignments of V [33] and C2 (PDB code 2LE9) domains and bacterial thioredoxin [45], [46], were made using CARA [47].

NMR titration experiments were performed to study interactions between RAGE domains and PAs #44 or #55, and #103. 200 µM of unlabeled PA #44 or #55 in NMR buffer were titrated into 50 μM [*U-* ^15^N] VC1 domain in four steps to yield VC1 domain to aptamer molar ratios of 2∶1, 1∶1, 1∶2 and 1∶4 respectively. The same procedure was followed to titrate unlabeled 200 μM PA #103 into a solution of 50 μM [*U-* ^15^N]-labeled C2 domain The results of the titration were monitored by ^1^H{^15^N}-HSQC. Over the course of titration, the signal to noise ratio of the peaks that did not show any changes was kept constant by adjusting the number of scans. We also titrated 100 μM unlabeled C2 domain in NMR buffer into 50 μM of [*U-* ^15^N] PA #103 in two steps to yield PA #103 to C2 domain molar ratios of 2∶1and 1∶2 respectively. The Thioredoxin scaffold was used as a negative control during titration experiments. No changes in the ^1^H{^15^N}-HSQC spectra of [*U-* ^15^N] VC1 domain or [*U-* ^15^N] C2 domain were observed after adding 200 μM of unlabeled thioredoxin scaffold, suggesting that thioredoxin does not bind to the RAGE domains.

### Docking studies

HADDOCK [48] was used to dock RAGE-C2 and anti-C2 PA #103 by using the previously determined structure for RAGE-C2 (Protein Data Bank entry 2ENS) and a model of PA #103 (**Table S2**). Chemical shift perturbations observed upon complex formation were used to define ambiguous interaction restraints (AIR) for residues at the interface (**Table S3**). Active residues were defined as those having either chemical shifts changes larger than 0.05 ppm or peak broadening of more than 30% compared to free protein. Passive residues were defined as all of the residues surrounding the interaction surfaces. AIRs were defined between every active residue of the first protein and all active and passive residues of the second protein and vice versa. A total of 5000 rigid-body docking trials were carried out using the standard HADDOCK protocol. The 100 lowest-energy solutions were used for subsequent semi-flexible simulated annealing and water refinement. The ten lowest-energy structures were used to represent the complex. Validation of the structures was performed with PROCHECK [43].

### Cell lines

Wild-type murine primary vascular smooth muscle cells were isolated and cultured from the aortas of 10-week-old male C57BL/6 mice (The Jackson Laboratory) using a modification of the procedure of Tarvo and Barret [49]. SMCs were cultured following an explant protocol in accordance with institutional guidelines. Cultures were composed of 95% SM-α-actin positivity based on immunostaining. Rat C6 glioma cells were obtained from ATCC (CCL-107) and maintained in Dulbecco's modified Eagle's medium supplemented with 10% fetal bovine serum (Invitrogen).

### Stimulation assay

Primary vascular smooth muscle cells and C6 glioma cells were seeded at 1×10^6^ cells/100 mm dish in complete medium and grown for 24 h before subjecting them to overnight starvation in serum-free medium. The next day cells were pre-incubated with domain specific PAs (3 μM) or controls (WT Thioredoxin or TSc with no peptide aptamer loop) for 30 min at 37°C, and stimulated with 10 μg/mL of S100B-BSA for five min. Cells were rinsed with ice-cold PBS and lysed using lysis buffer (Cell Signaling Technology) containing 1 mM phenylmethylsulfonyl fluoride and Complete Protease Inhibitors (Roche Applied Science).

Total cell lysate was immunoblotted and probed with ERK or pERK-specific antibodies (Cell Signaling Technology). HRP-conjugated donkey anti-rabbit IgG (Amersham Pharmacia) or HRP-conjugated sheep anti-mouse IgG (Amersham Pharmacia) were used to visualize specific bands. After probing with the anti-pERK antibody, membranes were stripped of bound immunoglobulins and re-probed with ERK antibody to assess total protein. Blots were scanned on an AlfaImager TM 2200 scanner equipped with AlfaEase (AlfaImager) FC 2200 software. Results are reported as a ratio of test antigen to relative total protein. In all Western blot studies, at least three cell lysates per group were used; results of representative experiments are shown.

## Results

### Construction of the library (prey) vector pLib2

**Figure 1:**
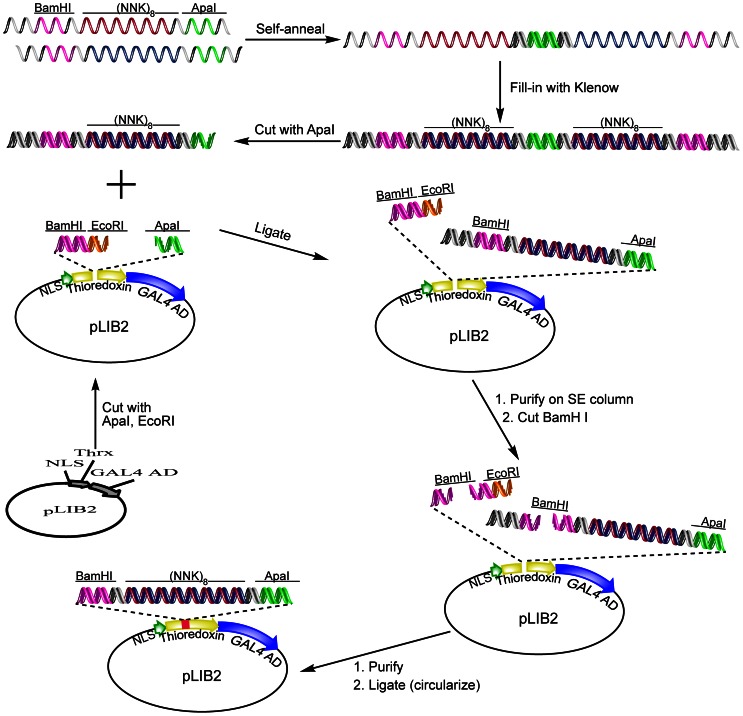
General scheme for cloning random inserts into the yeast-two-hybrid library vector. Two partially complementary strands containing randomized (NNK)_8_ nucleotide triplets (N- any nucleotide, K – G or T) are self-annealed, and the 5′ and 3′ ends are filled with Klenow. After cutting with ApaI, the duplex is ligated into plasmid pLIB2, which was pre-cut with ApaI and EcoRI. The ligation product is purified on a size exclusion column, cut with BamHI and circularized to reconstitute CLIPs. Cut sites are labeled. *NLS* is a nuclear localization sequence; *GAL4AD* is the GAL4 activation domain sequence.

*E.coli* Thioredoxin (TrxA) was selected as a stable protein scaffold to constrain the combinatorial peptide sequences that comprised the peptide aptamer (PA) library [18]. The TrxA reaction center was modified by introducing a polylinker site (BamHI, EcoRI, ApaI) (**Figure 1**), which simplifies directional cloning of peptide sequences and reduces the likelihood of PA aggregation by eliminating the cysteine residues that give rise to intra- and intermolecular disulfide bonds [17]. The modified TrxA scaffold was positioned upstream of the Gal4 activation domain in the Y2H library vector, pGADT7-AD, to yield pLib2. This arrangement eliminates the expression of truncated prey [17] due to the presence of stop codons in the random aptamer sequence. A number of amino acid substitutions aimed at enhancing PA solubility and folding properties were introduced in the later stages of scaffold optimization (see below). Although these substitutions could be introduced into the library vector, it is not absolutely necessary since, during Y2H selection, PAs are expressed as fusions of Gal4-AD, a very soluble protein that mitigates the potential low solubility of the aptamers.

### Construction of the random PA library

To build the PA library, oligonucleotide sequences containing 24 random nucleotides flanked by ApaI and BamHI sites, were synthesized (**Figure 1**). The random sequences were generated by using the degenerate codon, NNK, where N represents a mixture of equal amounts of adenine, thymine, guanine, and cytosine nucleotides and K represents a mixture containing equal amounts of thymine and guanine nucleotides. Using the degenerate codon to construct the library reduces the genetic code from 64 to 32 codons while coding for all 20 amino acids, and eliminates 2 of 3 stop codons [18], which minimizes the occurrence of truncated PAs.

Primer extension was used to build oligonucleotide duplexes containing random sequences [50]. Introducing priming sequences into the oligonucleotide design results in the addition of several extra amino acid residues to the aptamer loop at the TrxA reaction center, possibly destabilizing the TrxA scaffold. Restriction digestion of oligonucleotide duplexes to remove priming sequences results in a mixture of hard to separate products; unless removed from the subsequent ligation reaction, these products will compete with random duplex for vector sites and reduce the overall ligation efficiency [50]. To address this problem, we developed a new design for random oligonucleotides that incorporates a self-annealing stretch built around the GC rich ApaI restriction site (**Figure 1**). This design eliminates the formation of small restriction digestion reaction products, facilitating a two-step cloning scheme [50] that assures high-efficiency directional ligation, the absence of multiple inserts, and the construction of a very high quality PA library containing a minimal number of aberrant inserts.

**Figure 2:**
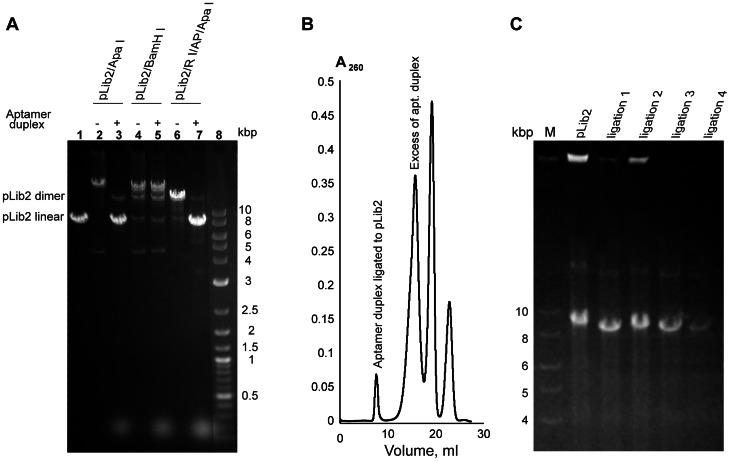
Construction and amplification of CLIPs. *****A*.**** Test of pLib2 vector self-ligation alone (lane 2) or in the presence of a 1000-fold excess of random PA DNA duplex digested with Apa I (lane 3). Excess PA does not affect the self-ligation of vector with incompatible ends (BamHI) (lanes 4 and 5). When pLib2 is digested with EcoRI, dephosphorylated with Antarctic Phosphatase (AP) and digested again with ApaI, the ligation reaction goes almost to completion with negligible amounts of vector self-ligation products formed (lanes 6 and 7). RI– EcoR I, AP– Antarctic Phosphatase. Lane 1 is linearized pLib2 plasmid and lane 8 is DNA molecular weight markers. ***B*** **.** Typical separation profile of pLib2/Apa I + PA duplex ligation products from excess duplex and other reactants on a Sephacryl S500 column. ***C*** **.** Example of amplification of ”recircularization” ligation (see text) with Phi 29 polymerase. Ligation reactions were split into smaller batches, amplified with Phi 29 and digested with BamHI (Lanes 3–6, respectively). Phi 29 amplification depends strongly on the quality of circular template and can fail if the yield of circular ligation product is low (Lane 6). Plasmid pLib2 was amplified under the same conditions and served as a control (Lane 2).

The general cloning scheme is outlined in **Figure 1**. To introduce a sequence coding for random octapeptides, (NNK)_8_, into the library vector, the oligonucleotide sequences, which contain complementary regions (**Table S1**), are self-annealed. Single-stranded regions are filled-in by using Klenow enzyme to generate the full duplex (6), which is digested with ApaI. The pLib2 vector is prepared by cutting with EcoRI, dephosphorylating the ends and digesting a second time with ApaI (**Figure 1**, **Figure 2A**). Duplex is used in 1000-fold molar excess in the first ligation reaction to suppress vector oligomerization and to ensure the completeness of reaction [50]. The ligation mix is purified by size exclusion chromatography to remove non-ligated oligonucleotide duplex (**Figure 2B**). The vector is digested with BamHI, re-purified, diluted to <5 ng/µL and allowed to self-ligate (circularize) in the presence of ligase, thus creating the Combinatorial Library of Improved Peptide aptamers, CLIPs (**Figure 2C**).

### Amplification of the CLIPs

Due to the inherent low efficiency of DNA uptake by cells [51], the only way to translate high library diversity into a large number of colonies on screening plates is to use milligram quantities of DNA in the transformation reaction. To generate such large amounts of DNA, the input CLIPs was first amplified *in vitro* to produce hundreds of micrograms of plasmid DNA, then further amplified by propagation in *E. coli*.

Phi29 DNA polymerase [52], [21], which has a strong preference for amplifying circular DNA, high processivity and an exceptionally low error rate, was used for multiple displacement amplification. In this method, random hexameric primers bind to multiple sites on a circular template to achieve exponential target amplification [52] (**Figure S2**). The input CLIPs was incubated with Phi29 for 15 hours at 30°C, with fresh polymerase/dNTP/random primer mix added every 3 hours [21]. The resulting mix was digested with BamHI (**Figure S2**) and purified by size exclusion chromatography. The total yield of purified CLIPs was ∼800 μg, providing an ∼2400-fold amplification of the library. The resulting DNA was re-circularized at low concentration. The efficiencies of test transformations were ∼10^8^ cfu/μg. Random colony PCR showed the presence of correct size inserts in 80% of the clones. Sequence analyses confirmed that Phi29 does not introduce mutations or rearrangements into the DNA constructs [21].

To acquire enough material for yeast transformation, the pool of CLIPs DNA was subjected to amplification in *E.coli*. Polymerase-amplified CLIPs was transformed into *E.coli* host strain DH10B by electroporation. Preparative scale isolation yielded several milligrams of plasmid DNA per experiment, corresponding to an additional 1000-fold amplification. Each transformation produced 10^8^–10^9^ unique clones, which was determined by plating small aliquots on appropriate selection plates. Although some of the library diversity was inevitably lost through this process, repeated transformation and preparation of plasmid DNA allowed us to create CLIPs with a combined titer of more than 3×10^10^ unique clones and sufficient DNA to ensure the transfer of this diversity into the yeast cells.

### Y2H library screening and confirmation of positive interactors

RAGE is a pattern recognition receptor that binds to classes of molecules rather than specific molecular structures [36], [53]. RAGE contains three extracellular immunoglobulin-like domains, V, C1, and C2, a trans-membrane helix and a short, 32 aa, intracellular tail indispensable for RAGE signaling [24], [54]. A soluble form of RAGE, sRAGE, consists of only the V, C1 and C2 domains. Since RAGE is a multiligand receptor, we hypothesized that RAGE signaling can be allosterically regulated by PA binding to any of the three extracellular domains. To test this assumption, four baits representing the V, C1, C2 and sRAGE fragments of human RAGE were cloned into the yeast shuttle vector, pGBKT7. The resulting constructs express a Gal4 binding domain-RAGE fragment fusion protein suitable for use as bait in a Y2H screen.

Mating of subsets of yeast containing CLIPs prey, ∼2×10^8^ clones, with yeast containing different RAGE domain baits, generated diploid sets of 7–15×10^6^ clones each. The mating efficiency varied between 5 and 12 percent. Diploids were spread at the rate of 5–10×10^6^ Leu^+^ cells per selection plate (-Ade, -Trp, -Leu) and incubated for up to a week. Well-developed colonies from the CLIPs selection plates were re-streaked onto selection media to evaluate the relative strength of interaction based on the manifestation of four different markers: two metabolic reporters (*ADE2, HIS3*) that allow selected clones to grow without adenine (-Ade) and histidine (-His) [55], [56], an antibiotic reporter (*AURI-C*) that provides cell survival in the presence of the highly toxic drug aureobasidin A [57], and a colorimetric reporter (*MEL1*) that facilitates blue-white screening when using the chromogenic substrate X-α-Gal.

Clones exhibiting the most proliferation on (-Ade, -Trp, -Leu), (+Aureobasidin A,-Trp, -Leu), (-His, -Trp, -Leu), and the most staining on (+X-α-Gal,-Trp, -Leu) plates (**Figure S3**) were selected for further analysis by ELISA. In this experiment, the *in vitro* binding of peptide aptamer-Gal4AD-HA epitope fusion proteins from yeast extracts, to bacterially expressed RAGE VC1 and C2 domains, was evaluated. Five PAs against the V domain, ten against the C1 domain, and four against the C2 domain were selected, and their sequences were determined (**Figure S4**).

### Improving the solubility and aggregation properties of the Thioredoxin scaffold

To over-express and purify ELISA-selected PAs from *E. coli*, the corresponding DNA fragments, obtained by yeast colony PCR, were re-amplified using a different set of oligonucleotide primers (**Table S1**) and cloned into bacterial vector pBAD (**Figure S1**). Mostly insoluble PAs were isolated. Generally, the solubility and aggregation properties of PAs depend on the aptamer loop sequence. Proteins with polar or negatively charged residues in the loop tend to be more soluble, while an excess of hydrophobic or positively charged residues commonly results in highly aggregated insoluble products. Since the presence of unfolded or misfolded species is often cited as one of the major factors causing protein aggregation, we attempted to stabilize the Thioredoxin Scaffold (TSc) perturbed by the insertion of an aptamer loop (**Figure S5**) by targeted mutagenesis of selected residues.

**Figure 3:**
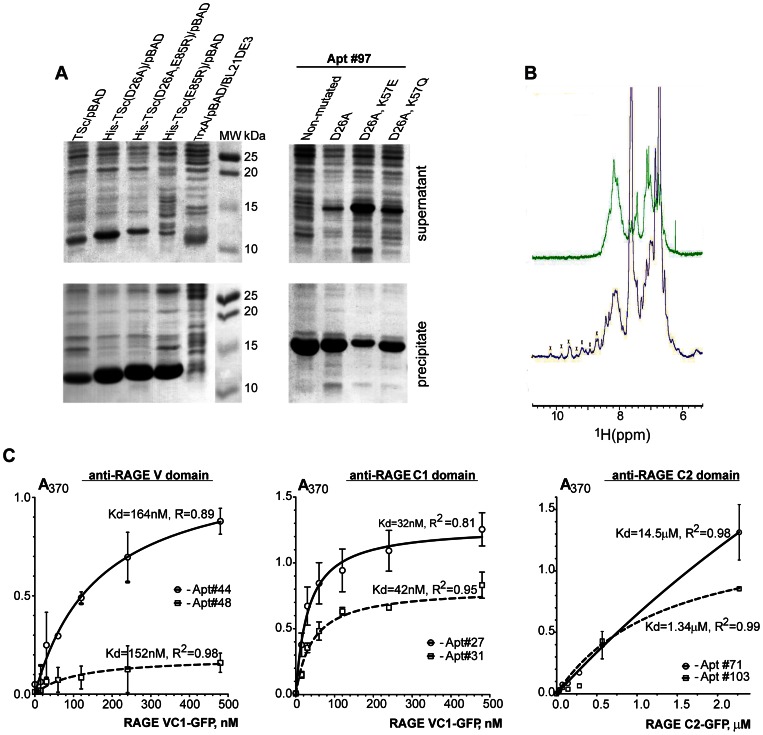
Expression and characterization of PAs. ***A*.** Effect of Thioredoxin scaffold (TSc) mutations on the solubility of PAs. Native TSc, PA #97, and PA #97 in the modified TSc were cloned into pBAD and overexpressed in *E. coli* BL21 (DE3) cells. After cell lysis, soluble (upper panels) and insoluble (lower panel) fractions were analyzed by SDS-PAGE. Note the increase in supernatant fraction of D26A-TSc (left upper gel panels), and increased PA solubility with K57E and K57Q mutations (right upper gel panel). ***B*** **.** ^1^H-NMR spectrum of PA #27 in wild type (green) or D26A TSc (purple). Limited dispersion in the amide region from 8.5 ppm to 7.5 ppm for the native TSc suggests either unfolded protein, or the existence of a large oligomer in solution. D26A-TSc, on the contrary, displays well dispersed amide peaks in the NMR spectrum region from 10 ppm to 6 ppm, implying a well-folded, predominantly monomeric solution structure (the apparent transverse relaxation time, T_2_, for amide ^1^H atoms of D26A-TSc at 20°C is 35 ms). Peaks arising from the folded conformation of D26A-TSc are marked with crosses. ***C*** **.** Binding curves for the binding of PAs to different RAGE-GFP fusion proteins. ELISA endpoint assays were run in triplicate. K_d_s were estimated using a model that assumed one strong binding site. SEM values are plotted for each data point (see text for details).

Aspartate 26 to alanine mutation, D26A, removes the negative charge buried at the bottom of hydrophobic cleft and provides a marked increase in protein solubility while retaining the structural fold [58], [59] (**Figure S5**). Another mutation, E85R, is based on the structure of homologous Thioredoxin from the thermophile *B. acidocaldarius* [60]. Mutation D26A increases the solubility of both the scaffold and the PA, while the E85R substitution has little effect (**Figure 3A**). PAs with a D26A-mutated scaffold exhibit increased solubility, better stability in solution, and better amide dispersion in 1D NMR spectra than wild type, reflecting the properly folded structure (**Figure 3B**).

To further improve solubility, we selected the two particularly insoluble anti-RAGE C2 domain PAs, #97 and #103, and conducted another round of scaffold modifications (**Figure 3A** **, Figure S5C**). We either removed (K57Q) or reversed (K57E) the positive charge of the buried K57 residue, which in wild-type Thioredoxin is involved in a salt-bridge with D26 [61], [62]. These mutations were designed to compensate for the removal of the negative charge in D26A. Results of mutant expression (**Figure 3A** **, Figure S5C**) indicate the D26A, K57E and, to a lesser extent, the D26A, K57Q double mutations markedly increase the in-cell solubility of both PAs #97 and #103. We incorporated the D26A and K57E mutations into the Thioredoxin Scaffold (TSc) for bacterial cloning, over-expression, and *in vitro* and in-cell testing of PAs.

### Binding of anti-RAGE peptide aptamers

The purity of over-expressed anti-RAGE PAs was verified by using gel electrophoresis, anion-exchange and size exclusion chromatography (**Figure 3A** **and Figure S5C**). The proper folding state was confirmed by acquiring 1D NMR amide dispersion spectra (**Figure 3B**). The apparent NMR transverse relaxation time, T_2_, for amide ^1^H atoms of anti-RAGE PAs at 20°C was ∼35 ms suggesting a monomeric state for the proteins. ELISA assays and NMR titrations of RAGE domains with [*U*-^15^N] PAs were used to evaluate binding affinities (**Figure 3C**). Since a relatively small subset, ∼0.05%, of the CLIPs was screened (extensive library screening was outside the scope of this research), only interactors with nanomolar to micromolar affinities were obtained. The equilibrium constants (K_d_) estimated from analyses of both NMR and ELISA results were in qualitative agreement (**Figure 3C**, see below). Anti-RAGE C1 domain PAs bound most strongly with a K_d_ of ∼10^−8^ M, anti-V domain binders exhibited a K_d_ of ∼10^−7^ M, and the affinities of anti-C2 domain aptamers were in the micromolar range.

### Structural characterization of anti-RAGE peptide aptamers in vitro

NMR spectroscopy provides a powerful way to identify interaction surfaces between proteins by using the chemical shift perturbation technique [63]. Chemical shifts are highly sensitive to changes in the electronic structure caused by molecular binding. The interface between two interacting proteins can be identified by changes in the position or intensity of the NMR resonances due to protein complex formation. To reduce spectral complexity, only one protein in the complex is labeled with ^15^N or ^13^C. In the ensuing ^15^N or ^13^C heteronuclear single quantum coherence (HSQC) experiments, only resonances from the labeled protein are represented in the NMR spectrum leaving the interacting partner cryptic.

**Figure 4:**
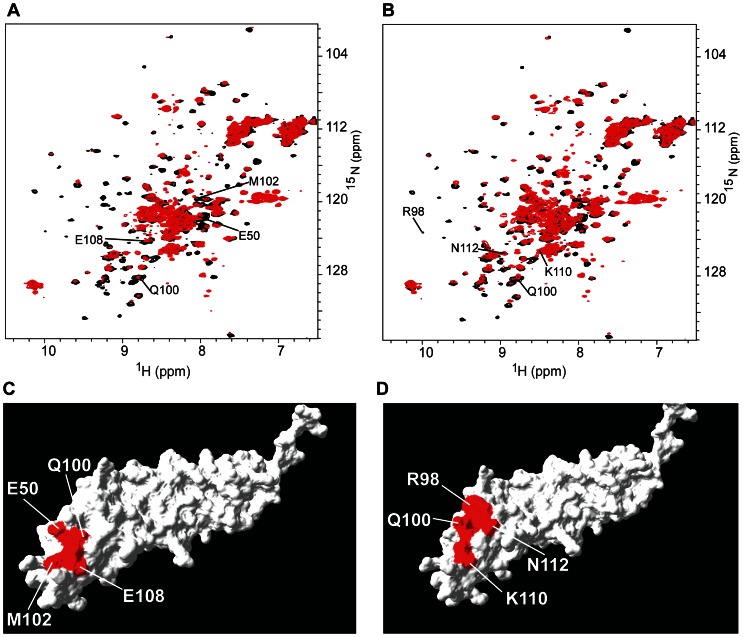
RAGE VC1 domain discriminates between V domain-targeted PAs #44 and #55. ***A*** **.** Overlay of ^1^H{^15^N}-HSQC spectra of free (black) and PA #44-bound (red) [*U-* ^15^N] RAGE VC1 domain. ***B*** **.** Overlay of ^1^H{^15^N}-HSQC spectra of free (black) and PA #55 bound (red) [*U-* ^15^N] RAGE VC1 domain. Extensive broadening of the NMR spectra indicates complex formation. Only V domain assignments are labeled. ***C*** **.** Space-filling model of the interaction surface of the PA #44-bound VC1 domain. The interacting surface of the V domain is shown in red. ***D*** **.** Space-filling model of the interaction surface of the PA #55-bound VC1 domain. The interacting surface is shown in red. Only cross-peaks that exhibited large changes are labeled. Space-filling models were based on the crystal structure of VC1 (PDB code 3CJJ) [29] and were prepared by using SWISS-PDB Viewer [68].

Anti-V domain PAs #44 and #55 were titrated into a solution containing [*U-* ^15^N] VC1 domain and changes in the resonance peaks of the backbone amide protons and nitrogens of VC1 residues were monitored to identify the interaction sites (**Figure 4A and 4B** **, Figures S6 and S7**). For both of these PAs, binding led to uniform broadening of the majority of the VC1 peaks, and differential broadening and chemical shift changes for a select set of V domain peaks (**Figure 4**, **Figures S6, and S7**). These changes are characteristic of a large, ∼36 kDa protein complex (MW of the VC1 domain is 24 kDa and the MW of a PA is 12 kDa) that undergoes intermediate to slow exchange between free and bound states with a K_d_ in the nM to sub-μM range [31], [63].

We identified the VC1 residues with more than 80% differential broadening and/or larger than a 0.05 ppm change in chemical shift as being involved in the interaction between the PA and RAGE (**Figures S6, S7**). PA #44 binds to a contiguous surface on VC1 consisting of E50, Q100, M102, and E108 (**Figure 4**). PA #55 also binds to a contiguous surface on VC1 consisting of residues R98, Q100, K110, and N112 (**Figure 4**). Importantly, the binding sites for both PAs are close to the S100B interaction surface on the V domain [29], which includes R98, E108 and K110, suggesting the possibility that the S100B-RAGE interaction can be directly blocked by PA binding.

**Figure 5:**
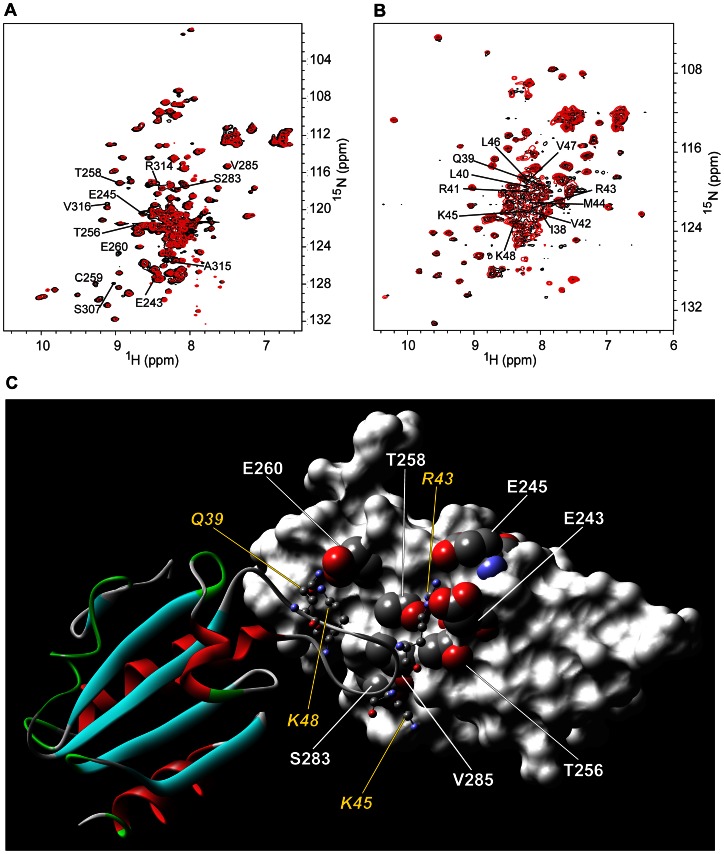
RAGE-C2 domain interacts with the PA loop #103. ***A*** **.** Overlay of ^1^H{^15^N}-HSQC spectra of free (black) and PA #103-bound (red) [*U-* ^15^N] RAGE-C2 domain. ***B*** **.** Overlay of ^1^H{^15^N}-HSQC spectra of free (black) and RAGE-C2 domain-bound (red) [*U-* ^15^N] PA #103. Only cross-peaks that exhibited large changes are labeled. ***C*** **.** Model of RAGE C2 domain and PA #103 was generated by using the HADDOCK server [48]. Glutamic acid residues 243, 245 and 322 form negatively charged contact points that interact with positively charged Arginines 41, 43 and 45 (shown as ball-and-stick models) of the aptamer loop. The PA is presented as a solid ribbon with color indicating the secondary structure. Residues in the PA loop (Gln39-Lys48) and N-terminal His-tag (His2-His7) are shown as stick models with coloring by secondary structure. Amino acid residue numbers correspond to those on panels A and B.

To characterize an interaction surface on the C2 domain of RAGE, anti-C2 PA #103 was titrated into a solution of [*U-* ^15^N] C2 domain (**Figure 5A**). Changes in the chemical shifts and intensities of the peaks corresponding to E243, E245, T256, T258, C259, E260, S283, and V286, as well as S307, R314, A315, and V316 (**Figure S8**) were observed. All of these residues, except S307, R314, A315, and V316, form a contiguous surface on the C2 domain.

Titrating [*U-* ^15^N] PA#103 into a solution of unlabeled C2 domain revealed that only the PA loop, 38-IQLRVRMKLV (**Figure 5B**), is affected by the interaction. This result allowed us to build a model of the complex by using the HADDOCK docking program [48] (**Figure 5** **, Tables S2, S3**). A previously determined structure of the RAGE C2 domain (Protein Data Bank entry 2ENS) was used as input to the program. The structure of the second docking partner, PA #103, was based on *E.coli* Thioredoxin coordinates (PDB entry 2TRX [64]) [40]. The docking of the two structures revealed that, besides surface complementarity, the interaction is driven by an electrostatic attraction between E243, E245, and E260 of the C2 domain and R43, K45, and K48 of PA #103 (**Figure 5C**). Importantly, this aptamer binds to the segment of C2 domain, which is located away from the binding surface for the majority of VC1 domain RAGE ligands, and therefore cannot directly block ligand-receptor interactions.

### Anti-RAGE aptamers inhibit RAGE signaling

**Figure 6:**
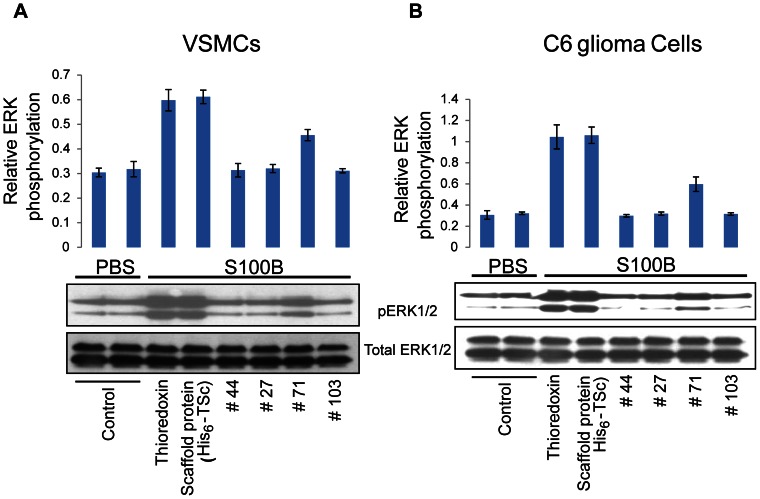
Aptamer binding inhibits RAGE signaling. Vascular smooth muscle cells (panel ***A***) and C6 glioma cells (panel ***B***) were pretreated with domain specific PAs or wild type Thioredoxin and TSc controls, and stimulated with S100B. Cell lysates were separated by SDS-PAGE and immunobloted with antibodies specific for phospho-ERK1/ERK2 (upper gel panels) or total ERK1/ERK2 (bottom gel panels). Images were digitized and results represented on the bar diagrams. Note the marked inhibition of ERK phosphorylation in the presence of anti-RAGE PAs. The data are representative of three separate experiments.

To test the functional implications of PAs, we used two RAGE-expressing cell types, C6 glioma cells and primary murine aortic vascular smooth muscle cells (VSMC) [25], [65] (**Figure 6**). Cells were activated with S100B, a known ligand of RAGE, in the presence of different PAs. The degree of activation was monitored by the increase in phosphorylation levels of proteins ERK1/2, signaling downstream of RAGE: [24]. Adding S100B to cells containing either wild type Thioredoxin or mutated TSc induces normal RAGE signaling in both C6 glioma and VSMC cells (**Figure 6**). Comparing lanes 3 and 4 with control lanes 1 and 2 in **Figure 6** suggests that the Thioredoxin platform used for PA construction does not affect RAGE signaling. Total ERK1/2 levels remained constant throughout all experiments. Adding S100B together with anti-V domain PA #44, anti-C1 domain PA #27 or anti-C2 domain PA # 103, markedly inhibited RAGE signaling. This strongly suggests that the binding sites masked by the PAs are critical for S100B-induced activation of RAGE. Anti C2 domain PA #71 only partially inhibited RAGE activation by S100B, possibly due to its weaker binding (**Figure 3C**). Interestingly, since the C2 domain is located away from the S100B binding site on RAGE, these results suggest that PAs #103 and #71 act as allosteric inhibitors of RAGE signaling.

## Discussion

We developed an efficient design for constructing a combinatorial library of improved peptide aptamers (CLIPs) for Y2H screening and used that library in this case study to identify PAs that alter signal transduction initiated by the binding of the physiological ligand S100B to the cell receptor RAGE. At the core of CLIPs technology is efficient directional cloning of randomized oligonucleotides within the context of the *thioredoxin* gene and amplification of the ligated library, which helps preserve the diversity by overcoming the limited transformation efficiency of the cells. The design is robust and has allowed us to build a library of 3×10^10^ clones. A small subset of this library was tested in a Y2H screen to identify a number of candidates that bind to this important receptor. No limitations exist to expand the library size to full theoretical complexity for a random octapeptide library of 10^12^ clones.

The insertion of random aptamer loop sequences into the wild type Thioredoxin structure often leads to destabilization of the Thioredoxin fold, potentially causing PAs to oligomerize. To overcome this problem we introduced two mutations within the Thioredoxin platform that produced soluble monomeric PAs. Future peptide libraries can be constructed in this re-engineered thioredoxin platform. The D26A mutation was critical since it eliminated a buried negative charge in the wild type Thioredoxin but became a destabilizing factor in the engineered platform. The small size of the monomeric PAs allowed us to characterize interactions with target molecules at atomic resolution by using NMR spectroscopy and also allowed us to consider PAs as highly specific drug-like molecules rather than as large particles that bind non-specifically.

By using RAGE domains as targets in a yeast two-hybrid screen, we isolated several PAs that recognize all three RAGE immunoglobulin domains [24]. The interaction surface on the VC1 and C2 domains clearly showed PA binding to distinctly different sites, with affinities ranging from tens of nanomolar to micromolar. NMR analyses of the interaction surfaces between PAs and the C2 domain of RAGE allowed us to build a solution model of the complex. The small, ∼100 Å^2^, contact surface on the C2 domain highlights the drug-like properties of PAs.

The effect of PAs on S100B-induced RAGE signal transduction was examined in human cells (**Figure 6**). The phosphorylation of RAGE effectors was dramatically decreased by the binding of anti-V, PA #44, anti-C1,PA #27 and anti-C2, PA #71 and PA #103, domain PAs. PAs #27 and #44 recognize sites that are proximal to the S100B binding site [29], [30] (**Figure 6**, see also **Figure 4B**), suggesting that these PAs inhibit signaling by directly blocking the S100B-RAGE interaction. The sites for PAs #71 and #103 are located in the membrane proximal part of RAGE far from the S100B binding site [29], [30] (**Figure 6**, see also **Figure 5**), implying that this site may allosterically affect RAGE signaling.

It is important that, unlike *in vitro* selection, interactors discovered through Y2H screening do not compete with each other for target sites during selection [32]. As a result, the Y2H selection scheme can identify PAs that bind to different or even overlapping target sites without being biased by the highest affinity binders [66] (**Figure 4**). These binders can be used to explore the underlying functionality of biological networks, especially in receptor-dependent signal transduction pathways such as RAGE signaling [27], [30], or serve as guides to discover small-molecule drug candidates [67].

Most receptors involved in signal transduction undergo either homo- or hetero-oligomerization coupled with structural rearrangement of the domains. Conformational changes affect not only ligand proximal domains but also domains juxtaposed to the membrane and cytosolic part of the receptor. Due to the transient nature of these interactions, it is highly desirable to work with wild type biological systems, in which absolute and relative concentrations of the receptors are not perturbed by mutations and non-physiological over-expression of biologically active molecules. Monomeric PAs selected from CLIPs allow us to block specific sites on the target receptor without affecting other physiological interactions of the receptor. In this respect, CLIPs technology is similar to site-directed mutagenesis except that it does not require changes in the primary structure of the protein and can be performed at physiologically relevant concentrations. In summary, the availability of specific binders that recognize different sites on a target combined with structural biology studies and functional assays provides an opportunity to dissect complex biological pathways with minimum invasiveness.

## Supporting Information

Figure S1 **General scheme for recloning of PAs for bacterial expression.** A yeast colony, identified in Y2H screens and confirmed as a positive interactor, is used as a template for yeast colony PCR. The amplified DNA fragment is used to determine the sequence of the inserted octapeptide and re-amplified to prepare the cloning insert in one of two ways. To simultaneously introduce mutations that improve the characteristics of the PA, a fragment of the Thioredoxin scaffold containing the embedded PA sequence is amplified by using the corresponding oligonucleotides (Table S1). The amplified DNA is then used in overlap extension PCR cloning. Conventional re-cloning involves amplifying and attaching 5′ and 3′ restriction sites to the entire unmodified Thioredoxin scaffold (TSc), which contains an embedded PA loop. Fragments obtained this way are digested and ligated into the vector with compatible ends. Both schemes were employed at different stages of the research.
(DOCX)

Figure S2 **General scheme for Phi 29 amplification of CLIPs.** Random hexamer primers are annealed to pLib2. Plasmids are amplified by using Phi29 DNA polymerase forming concatameric DNA strands. The concatamers are digested with BamHI and purifed by using size exclusion chromatography. Self–ligation provides up to a 1000-fold amplification of CLIPs.
(DOCX)

Figure S3 **Library screening on different selection plates.** Diploids were initially selected on (-Leu, -Trp,-Ade) plates. Clones that demonstrated robust growth on (-Leu, -Trp, -Ade), (-Leu, -Trp, +Aureobasidin A), and (-Leu, -Trp), and staining on (-Leu, -Trp, -His, +X-α-Gal) plates were selected for further analysis.
(DOCX)

Figure S4 **Alignment of amino acid sequences of PA inserts generated against V domain (panel A), C1 domain (panel B) and C2 domain (panel C) of human RAGE.** PA numbers are given in the left columns. Homology sequence alignment was performed with the PRALINE software [69], residue coloring scheme is based on amino acid properties. Sequence diversity suggests that the selected PAs bind to distinct sites on their targets.
(DOCX)

Figure S5 **Summary of Thioredoxin scaffold mutations.** ***A*** **.** Solid ribbon representation of the reduced form of *E.coli* Thioredoxin (PDB entry 1XOB). Mutated amino acid residues are displayed as ball-and-stick models, corresponding ribbon sections are shown in red. ***B*** **.** Comparison of PA and wild type Thioredoxin sequences. Substitutions are marked by red circles. ***C*** **.** Effect of thioredoxin scaffold (TSc) mutations on the solubility of PA #103. PA #103 in the native and modified TSc were cloned into pBAD and overexpressed in *E. coli* BL21 (DE3) cells. After cell lysis, equal loads of soluble (upper panels) and insoluble (lower panel) fractions were analyzed by SDS-PAGE. Note the increased PA solubility with K57E and K57Q mutations (upper gel panel). Double mutations D26A, D15N and D26A, P76A resulted in the decrease in soluble fraction of PA #103.
(DOCX)

Figure S6 **Changes in the [** ***U-*** **^15^N] VC1 NMR signal intensities and chemical shifts due to binding to PA #44.** Unlabeled PA #44, from a 1 mM stock solution, was titrated into 100 μM [*U-* ^15^N] VC1 dissolved in NMR buffer (10 mM sodium phosphate, pH 7.5, 100 mM Na_2_S_2_O_3_, 0.02% (w/v) NaN_3_, 90%/10%H_2_O/D_2_O) to a molar ratio of 2∶1. The titration was monitored by collecting ^1^H{^15^N}-HSQC spectra. (**A**) VC1 domain NMR signal intensity changes were calculated by *ΔI  = (I_f_ – I_b_)/I_f_*, where *I_f(b)_* is the NMR signal intensity of free or PA #44-bound VC1. Most of the VC1 residues exhibited uniform broadening upon complex formation. Residues that exhibited signal broadening above 80% were considered to constitute the VC1 interaction surface. (**B**) VC1 domain chemical shift changes (Ω) were calculated by *Ω  = ((Δδ_H_)^2^ + (Δδ_N_/4)^2^)^1/2^*, where *Δδ_H_* and *Δδ_N_* are the changes in amide proton and nitrogen chemical shifts, respectively. Residues that exhibited chemical shift changes above 0.06 ppm, were considered to constitute the VC1 interaction surface. Cut-offs for selecting residues involved in the interaction between VC1 and PA #44 are indicated by red arrows. Since PA #44 was selected against the V domain, only V domain residues are shown on the graphs.
(DOCX)

Figure S7 **Changes in the [** ***U-*** **^15^N] VC1 NMR signal intensities and chemical shifts due to PA # 55 binding.** Unlabeled PA #55, from a 1 mM stock solution was titrated into 100 μM [*U-* ^15^N] VC1 dissolved in NMR buffer (10 mM sodium phosphate, pH 7.5, 100 mM Na_2_S_2_O_3_, 0.02% (w/v) NaN_3_, 90%/10%H_2_O/D_2_O) to a molar ratio of 2∶1. The titration was monitored by collecting ^1^H{^15^N}-HSQC spectra. (**A**) VC1 domain NMR signal intensity changes were calculated by *ΔI  = (I_f_ – I_b_)/I_f_*, where *I_f(b)_* is the NMR signal intensity of free or PA#55-bound VC1. Most of the VC1 residues exhibited uniform broadening upon complex formation. Residues that exhibited signal broadening above 80% were considered to constitute the VC1 interaction surface. (**B**) VC1 domain chemical shift changes (Ω) were calculated by *Ω =  ((Δδ_H_)^2^ + (Δδ_N_/4)^2^)^1/2^*, where *Δδ_H_* and *Δδ_N_* are the changes in amide proton and nitrogen chemical shifts, respectively. Residues that exhibited chemical shift changes above 0.06 ppm, were considered to constitute the VC1 interaction surface. Cut-offs for selecting residues involved in the interaction between VC1 and PA #55 are indicated by red arrows. Since PA #55 was selected against the V domain, only V domain residues are shown on the graphs.
(DOCX)

Figure S8 **Changes in [** ***U-*** **^15^N] C2 NMR signal intensities and chemical shifts due to PA #103 binding.** Unlabeled PA #103, from a 1 mM stock solution, was titrated into 100 μM [*U-* ^15^N] C2 dissolved in NMR buffer (10 mM sodium phosphate, pH 7.5, 100 mM Na_2_S_2_O_3_, 0.02% (w/v) NaN_3_, 90%/10%H_2_O/D_2_O) to a molar ratio of 2∶1. The titration was monitored by collecting ^1^H{^15^N}-HSQC spectra. (**A**) C2 domain NMR signal intensity changes were calculated by *ΔI  = (I_f_ – I_b_)/I_f_*, where *I_f(b)_* is the NMR signal intensity of free or PA #103-bound C2. Most of the C2 residues exhibited uniform broadening upon complex formation. Residues that exhibited signal broadening above 25% were considered to constitute the C2 interaction surface. (**B**) C2 domain chemical shift changes (Ω) were calculated by *Ω =  ((Δδ_H_)^2^ + (Δδ_N_/4)^2^)^1/2^*, where *Δδ_H_* and *Δδ_N_* are the changes in amide proton and nitrogen chemical shifts, respectively. Residues that exhibited chemical shift changes above 0.01 ppm, were considered to constitute the C2 interaction surface. Cut-offs for selecting residues involved in the interaction between C2 and PA #103 are indicated by red arrows. The lower degree of signal broadening and chemical shift changes for the PA #103-C2 complex, as compared to PA #44-VC1, correspond to weaker binding (see Figure 3C).
(DOCX)

Table S1 **List of oligonucleotides used.**
(DOCX)

Table S2 **Structural Statistics from the HADDOCK restrained calculations of the C2–PA #103 complex.**
(DOCX)

Table S3 **HADDOCK AIRs restrains used to calculate C2-PA #103 complex.**
(DOCX)
