# Supplementary material for: Combinatorial Library of Improved Peptide Aptamers, CLIPs to Inhibit RAGE Signal Transduction in Mammalian Cells
Source: PLoS One. 2013 Jun 13;8(6):e65180. doi: 10.1371/journal.pone.0065180 (PMC3681763; doi:10.1371/journal.pone.0065180)
Supplement: Figure S1 — General scheme for recloning of PAs for bacterial expression. A yeast colony, identified in Y2H screens and confirmed as a positive interactor, is used as a template for yeast colony PCR. The amplified DNA fragment is used to determine the sequence of the inserted octapeptide and re-amplified to prepare the cloning insert in one of two ways. To simultaneously introduce mutations that improve the characteristics of the PA, a fragment of the Thioredoxin scaffold containing the embedded PA sequence is amplified by using the corresponding oligonucleotides (Table S1). The amplified DNA is then used in overlap extension PCR cloning. Conventional re-cloning involves amplifying and attaching 5′ and 3′ restriction sites to the entire unmodified Thioredoxin scaffold (TSc), which contains an embedded PA loop. Fragments obtained this way are digested and ligated into the vector with compatible ends. Both schemes were employed at different stages of the research. (DOCX) [file pone.0065180.s001.docx]

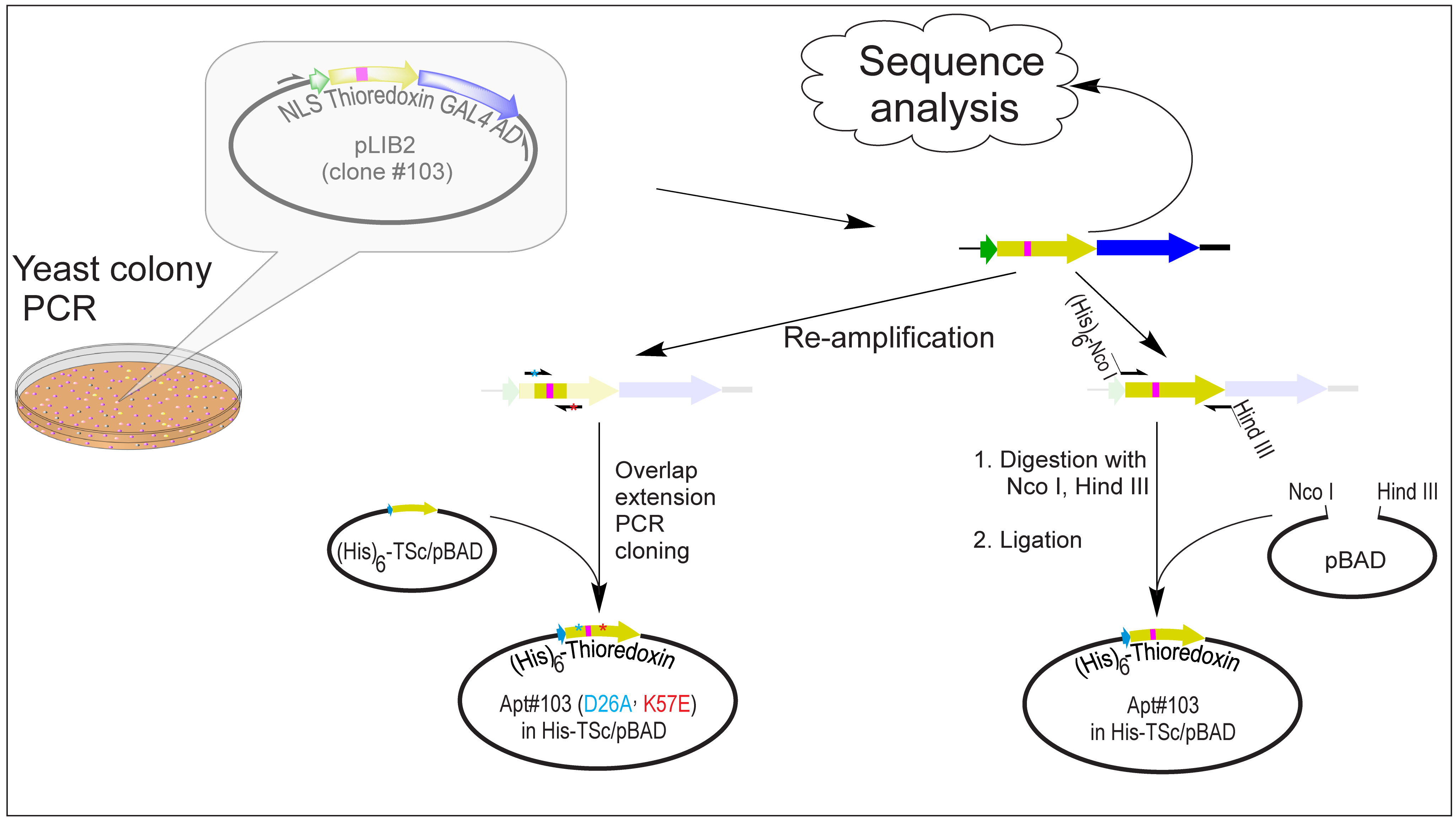


**Figure S1.** General scheme for re-cloning peptide PA inserts into bacterial expression vectors. A yeast colony, identified in Y2H screens and confirmed as a positive interactor, is used as a template for yeast colony PCR. The amplified DNA fragment is used to determine the sequence of the inserted octapeptide and re-amplified to prepare the cloning insert in one of two ways. To simultaneously introduce mutations that improve the characteristics of the PA, a fragment of the Thioredoxin scaffold containing the embedded PA sequence is amplified by using the corresponding oligonucleotides (Table S1). The amplified DNA is then used in overlap extension PCR cloning. Conventional re-cloning involves amplifying and attaching 5’ and 3’ restriction sites to the entire unmodified Thioredoxin scaffold (TSc), which contains an embedded PA loop. Fragments obtained this way are digested and ligated into the vector with compatible ends. Both schemes were employed at different stages of the research.
