# Supplementary material for: Combinatorial Library of Improved Peptide Aptamers, CLIPs to Inhibit RAGE Signal Transduction in Mammalian Cells
Source: PLoS One. 2013 Jun 13;8(6):e65180. doi: 10.1371/journal.pone.0065180 (PMC3681763; doi:10.1371/journal.pone.0065180)
Supplement: Figure S2 — General scheme for Phi 29 amplification of CLIPs. Random hexamer primers are annealed to pLib2. Plasmids are amplified by using Phi29 DNA polymerase forming concatameric DNA strands. The concatamers are digested with BamHI and purifed by using size exclusion chromatography. Self–ligation provides up to a 1000-fold amplification of CLIPs. (DOCX) [file pone.0065180.s002.docx]

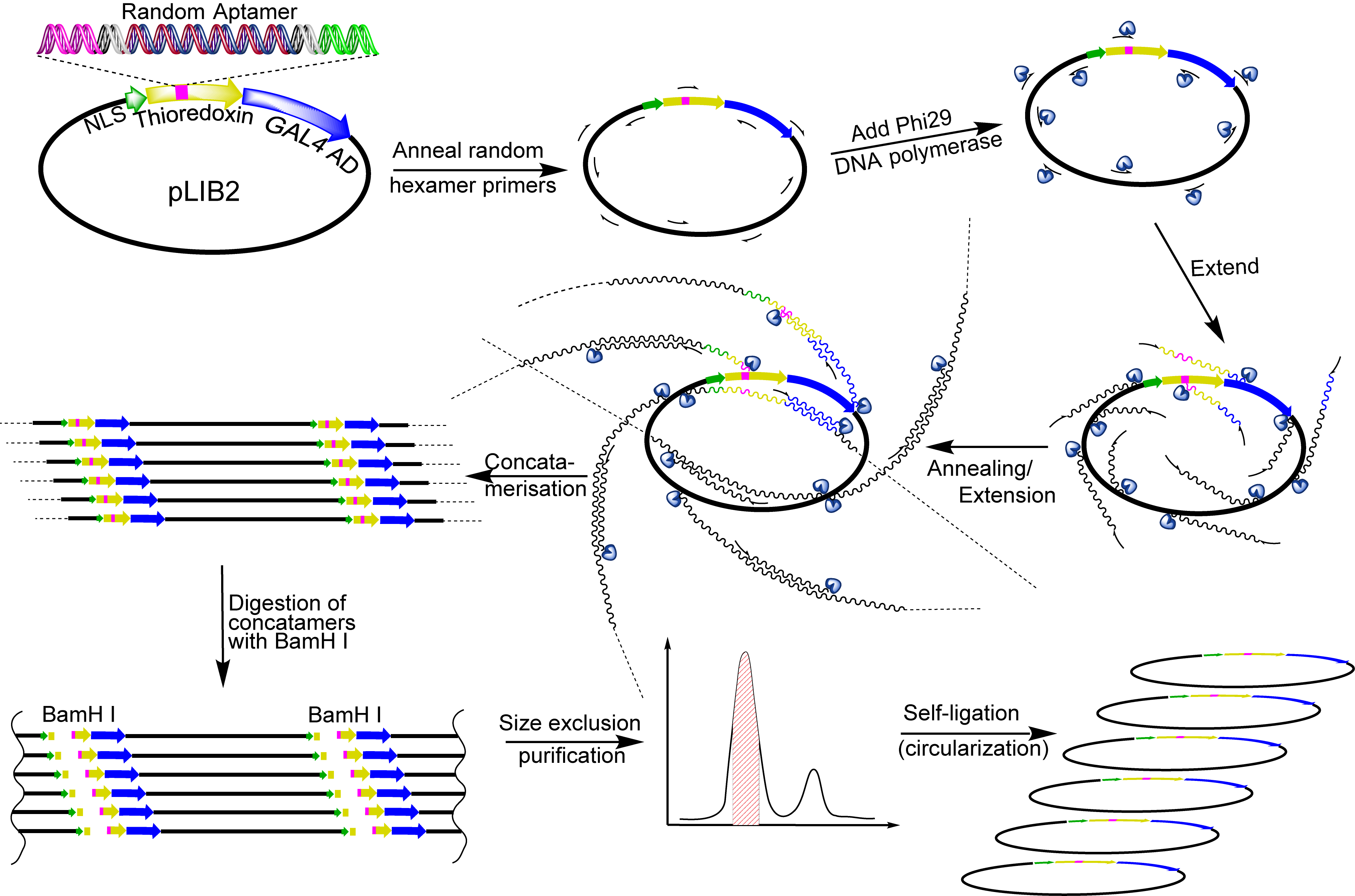


**Figure S2** General scheme for Phi 29 amplification of CLIPs. Random hexamer primers are annealed to pLib2. Plasmids are amplified by using Phi29 DNA polymerase forming concatameric DNA strands. The concatamers are digested with BamHI and purifed by using size exclusion chromatography. Self–ligation provides up to a 1000-fold amplification of CLIPs.
