# Supplementary material for: Combinatorial Library of Improved Peptide Aptamers, CLIPs to Inhibit RAGE Signal Transduction in Mammalian Cells
Source: PLoS One. 2013 Jun 13;8(6):e65180. doi: 10.1371/journal.pone.0065180 (PMC3681763; doi:10.1371/journal.pone.0065180)
Supplement: Figure S3 — Library screening on different selection plates. Diploids were initially selected on (-Leu, -Trp,-Ade) plates. Clones that demonstrated robust growth on (-Leu, -Trp, -Ade), (-Leu, -Trp, +Aureobasidin A), and (-Leu, -Trp), and staining on (-Leu, -Trp, -His, +X-α-Gal) plates were selected for further analysis. (DOCX) [file pone.0065180.s003.docx]

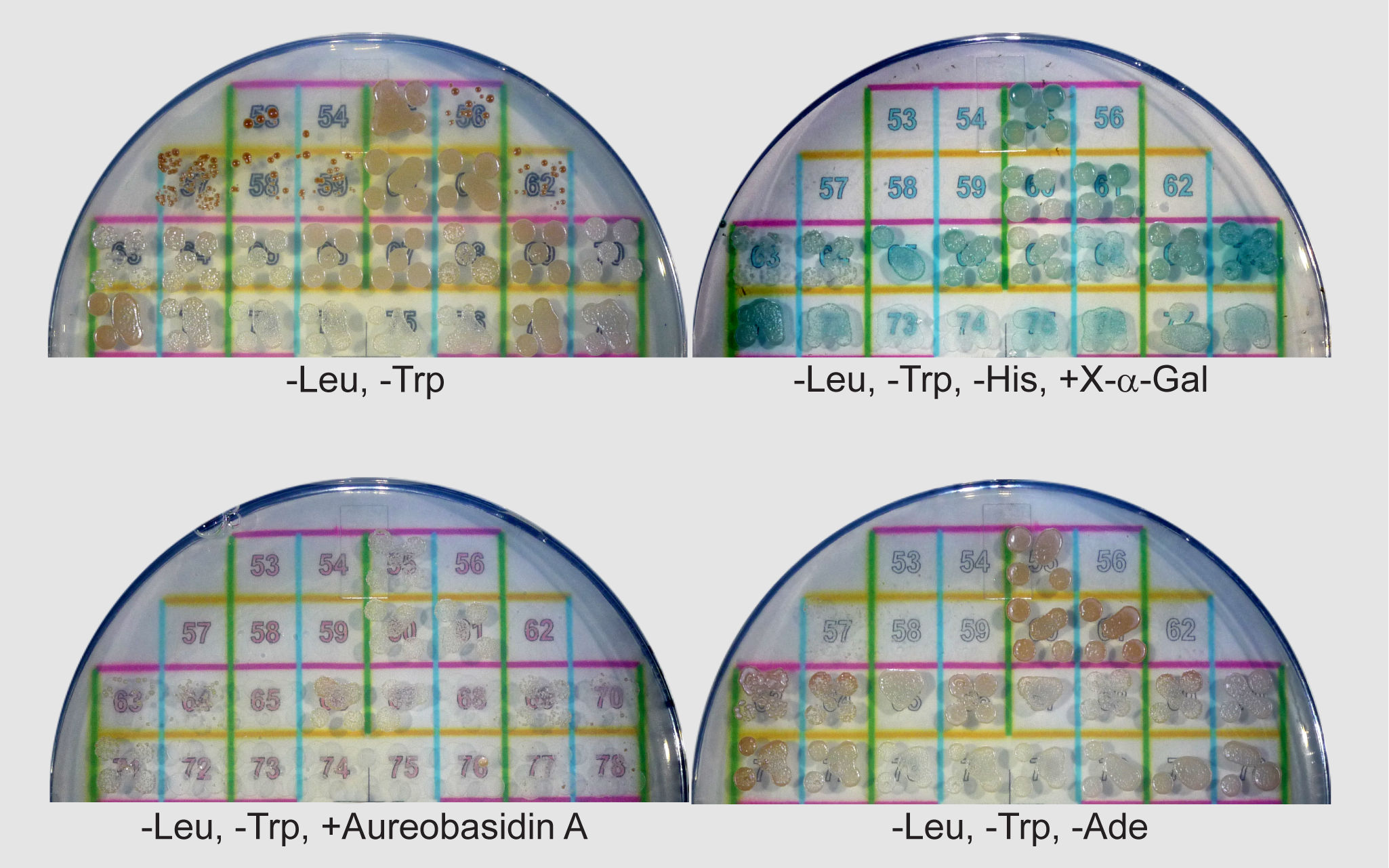


**Figure S3.** Library screening on different selection plates. Diploids were initially selected on (-Leu, -Trp,-Ade ) plates. Clones that demonstrated robust growth on (-Leu, -Trp,-Ade ), (-Leu, -Trp, +Aureobasidin A), and (-Leu, -Trp), and staining on (-Leu, -Trp, -His, +X-α-Gal ) plates were selected for further analysis.
