# Supplementary material for: Combinatorial Library of Improved Peptide Aptamers, CLIPs to Inhibit RAGE Signal Transduction in Mammalian Cells
Source: PLoS One. 2013 Jun 13;8(6):e65180. doi: 10.1371/journal.pone.0065180 (PMC3681763; doi:10.1371/journal.pone.0065180)
Supplement: Figure S4 — Alignment of amino acid sequences of PA inserts generated against V domain (panel A), C1 domain (panel B) and C2 domain (panel C) of human RAGE. PA numbers are given in the left columns. Homology sequence alignment was performed with the PRALINE software [69], residue coloring scheme is based on amino acid properties. Sequence diversity suggests that the selected PAs bind to distinct sites on their targets. (DOCX) [file pone.0065180.s004.docx]

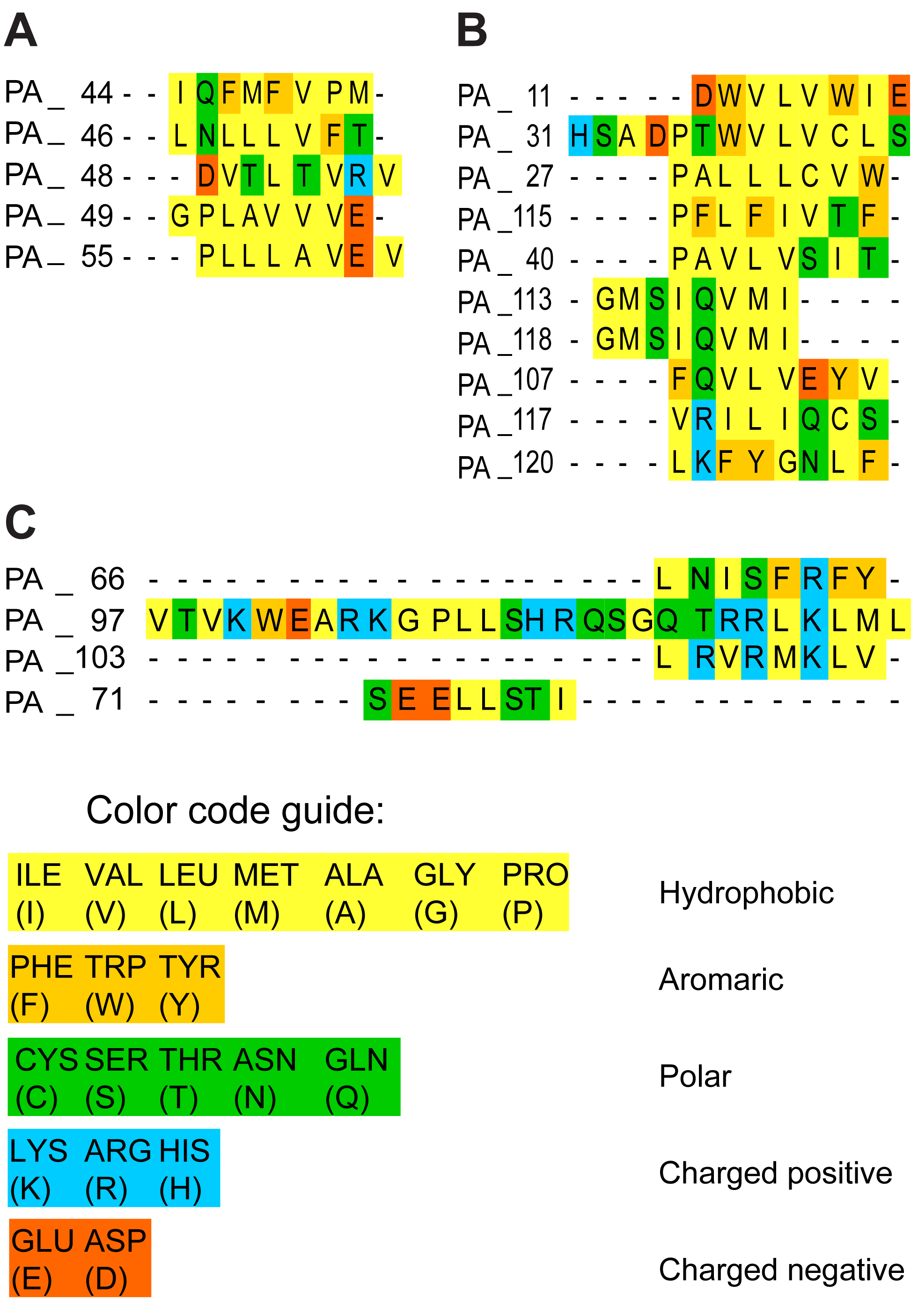


**Figure S4.** Alignment of amino acid sequences of PA inserts generated against V domain (panel A), C1 domain (panel B) and C2 domain (panel C) of human RAGE (PA numbers are given in the left columns). Homology sequence alignment was performed with the PRALINE software, residue coloring scheme is based on amino acid properties. Sequence diversity suggests that the selected PAs bind to distinct sites on their targets.
