# Supplementary material for: Combinatorial Library of Improved Peptide Aptamers, CLIPs to Inhibit RAGE Signal Transduction in Mammalian Cells
Source: PLoS One. 2013 Jun 13;8(6):e65180. doi: 10.1371/journal.pone.0065180 (PMC3681763; doi:10.1371/journal.pone.0065180)
Supplement: Figure S5 — Summary of Thioredoxin scaffold mutations. A . Solid ribbon representation of the reduced form of E.coli Thioredoxin (PDB entry 1XOB). Mutated amino acid residues are displayed as ball-and-stick models, corresponding ribbon sections are shown in red. B . Comparison of PA and wild type Thioredoxin sequences. Substitutions are marked by red circles. C . Effect of thioredoxin scaffold (TSc) mutations on the solubility of PA #103. PA #103 in the native and modified TSc were cloned into pBAD and overexpressed in E. coli BL21 (DE3) cells. After cell lysis, equal loads of soluble (upper panels) and insoluble (lower panel) fractions were analyzed by SDS-PAGE. Note the increased PA solubility with K57E and K57Q mutations (upper gel panel). Double mutations D26A, D15N and D26A, P76A resulted in the decrease in soluble fraction of PA #103. (DOCX) [file pone.0065180.s005.docx]

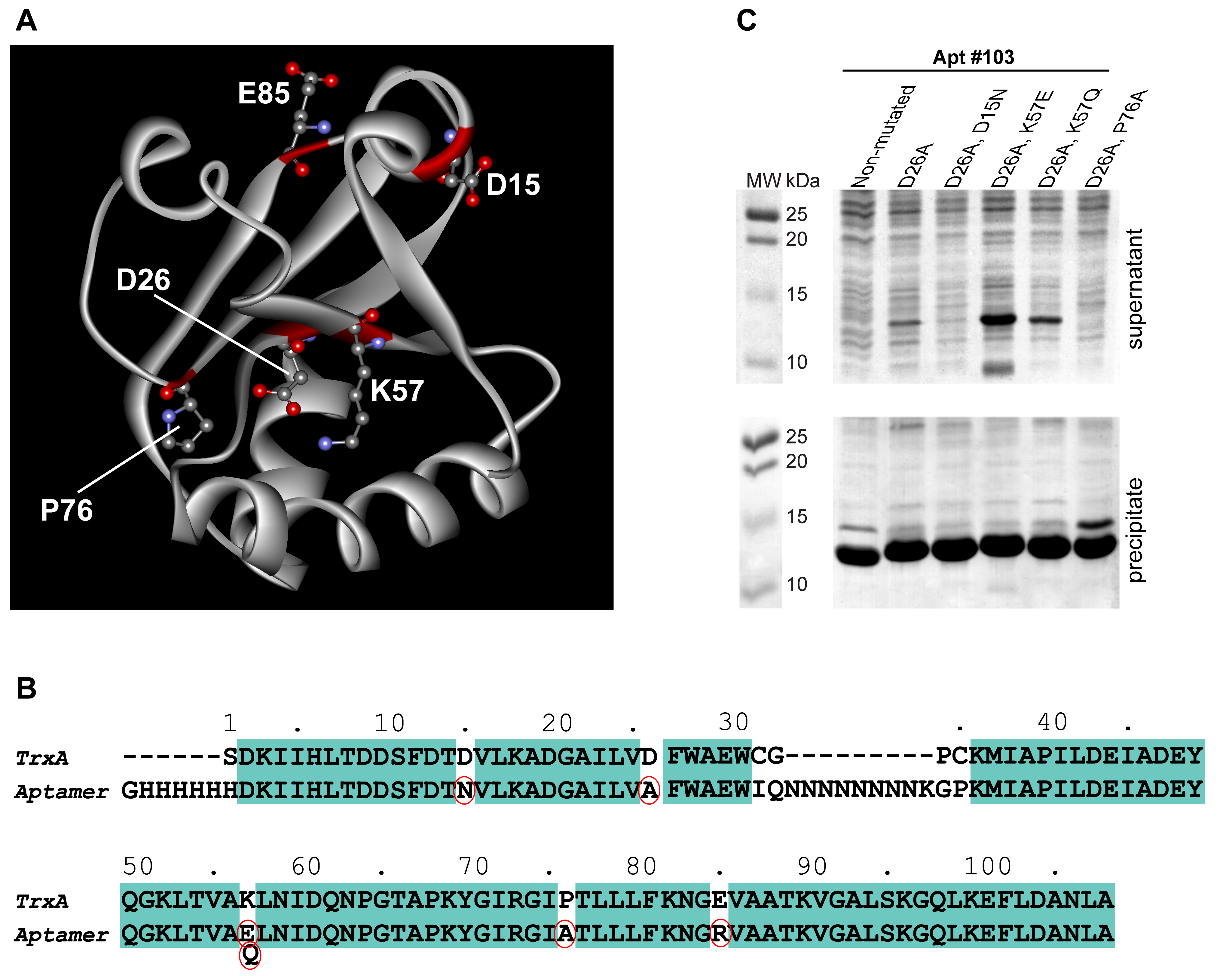


**Figure S5**. Summary of Thioredoxin scaffold mutations. ***A.*** Solid ribbon representation of the reduced form of *E.coli* Thioredoxin (PDB entry 1XOB). Mutated amino acid residues are displayed as ball-and-stick models, corresponding ribbon sections are shown in red. ***B.*** Comparison of PA and wild type Thioredoxin sequences. Substitutions are marked by red circles. ***C.*** Effect of thioredoxin scaffold (TSc) mutations on the solubility of PA #103. PA #103 in the native and modified TSc were cloned into pBAD and overexpressed in *E. coli* BL21 (DE3) cells. After cell lysis, equal loads of soluble (upper panels) and insoluble (lower panel) fractions were analyzed by SDS-PAGE. Note the increased PA solubility with K57E and K57Q mutations (upper gel panel). Double mutations D26A, D15N and D26A, P76A resulted in the decrease in soluble fraction of PA #103.
