# Supplementary material for: Combinatorial Library of Improved Peptide Aptamers, CLIPs to Inhibit RAGE Signal Transduction in Mammalian Cells
Source: PLoS One. 2013 Jun 13;8(6):e65180. doi: 10.1371/journal.pone.0065180 (PMC3681763; doi:10.1371/journal.pone.0065180)
Supplement: Figure S7 — Changes in the [ U- 15N] VC1 NMR signal intensities and chemical shifts due to PA # 55 binding. Unlabeled PA #55, from a 1 mM stock solution was titrated into 100 μM [U- 15N] VC1 dissolved in NMR buffer (10 mM sodium phosphate, pH 7.5, 100 mM Na2S2O3, 0.02% (w/v) NaN3, 90%/10%H2O/D2O) to a molar ratio of 2∶1. The titration was monitored by collecting 1H{15N}-HSQC spectra. (A) VC1 domain NMR signal intensity changes were calculated by ΔI = (If – Ib)/If, where If(b) is the NMR signal intensity of free or PA#55-bound VC1. Most of the VC1 residues exhibited uniform broadening upon complex formation. Residues that exhibited signal broadening above 80% were considered to constitute the VC1 interaction surface. (B) VC1 domain chemical shift changes (Ω) were calculated by Ω = ((ΔδH)2 + (ΔδN/4)2)1/2, where ΔδH and ΔδN are the changes in amide proton and nitrogen chemical shifts, respectively. Residues that exhibited chemical shift changes above 0.06 ppm, were considered to constitute the VC1 interaction surface. Cut-offs for selecting residues involved in the interaction between VC1 and PA #55 are indicated by red arrows. Since PA #55 was selected against the V domain, only V domain residues are shown on the graphs. (DOCX) [file pone.0065180.s007.docx]

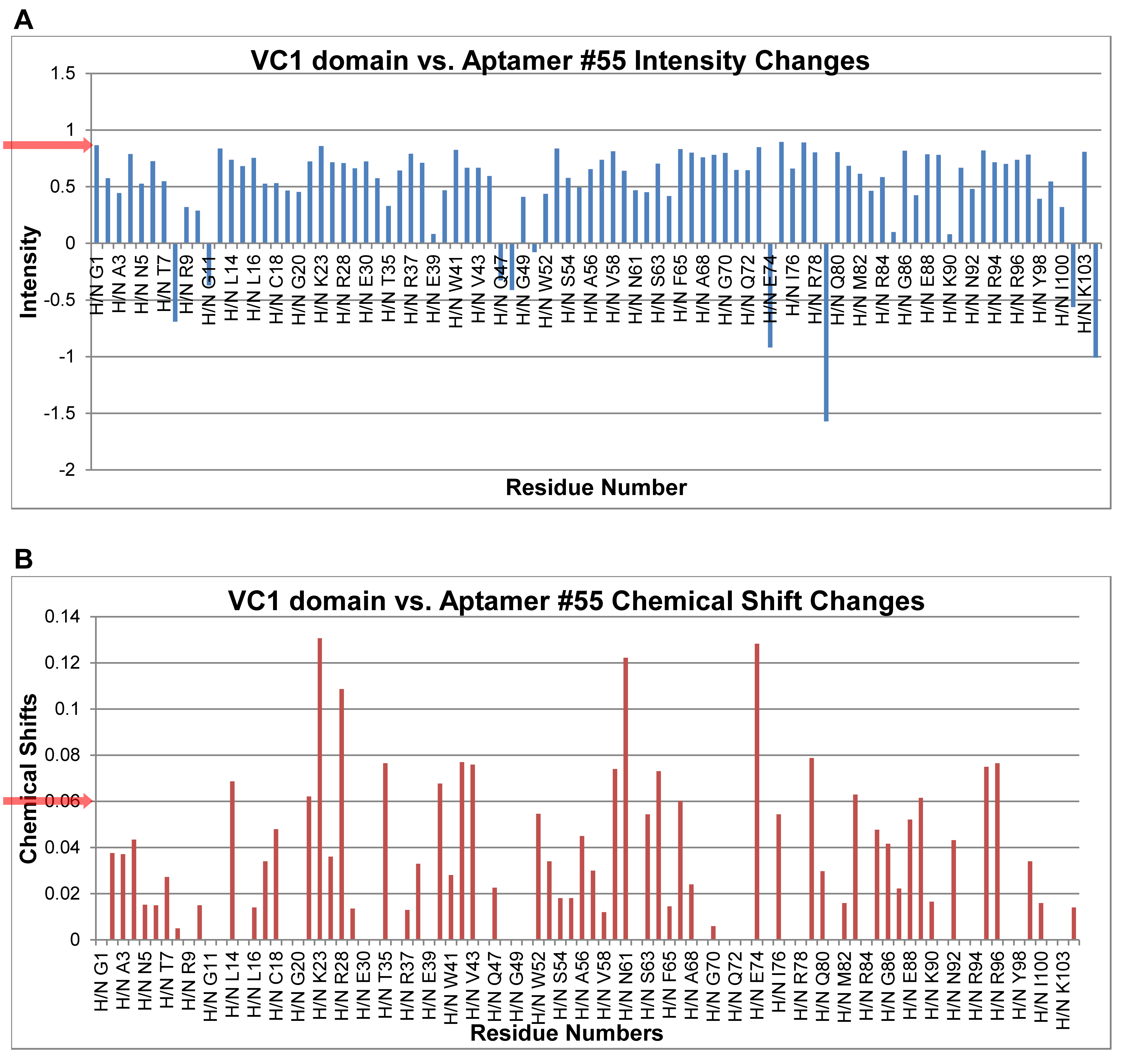


**Figure S7.** Changes in the [*U-*^15^N] VC1 NMR signal intensities and chemical shifts due to PA # 55 binding. Unlabeled PA #55, from a 1 mM stock solution was titrated into 100 μM [*U-*^15^N] VC1 dissolved in NMR buffer (10 mM sodium phosphate, pH 7.5, 100 mM Na_2_S_2_O_3_, 0.02% (w/v) NaN_3_, 90%/10%H_2_O/D_2_O) to a molar ratio of 2:1. The titration was monitored by collecting ^1^H,^15^N-HSQC spectra. (**A**) VC1 domain NMR signal intensity changes were calculated by

$$\Delta I=(I_{0}-I_{f})/I_{0}$$

where $I_{0(f)}$ is the NMR signal intensity of free or PA#55-bound VC1. Most of the VC1 residues exhibited uniform broadening upon complex formation. Residues that exhibited signal broadening above 80% were considered to constitute the VC1 interaction surface. (**B**) VC1 domain chemical shift changes (Ω) were calculated by

$$\Omega=\sqrt{\Delta\delta_{H}^{2}+(\Delta\delta_{N}/4)^{2}}$$

where $\Delta\delta_{H}$and $\Delta\delta_{N}$are the changes in amide proton and nitrogen chemical shifts, respectively. Residues that exhibited chemical shift changes above 0.06 ppm, were considered to constitute the VC1 interaction surface. Cut-offs for selecting residues involved in the interaction between VC1 and PA #55 are indicated by red arrows. Since PA #55 was selected against the V domain, only V domain residues are shown on the graphs.
