# Supplementary material for: Combinatorial Library of Improved Peptide Aptamers, CLIPs to Inhibit RAGE Signal Transduction in Mammalian Cells
Source: PLoS One. 2013 Jun 13;8(6):e65180. doi: 10.1371/journal.pone.0065180 (PMC3681763; doi:10.1371/journal.pone.0065180)
Supplement: Figure S8 — Changes in [ U- 15N] C2 NMR signal intensities and chemical shifts due to PA #103 binding. Unlabeled PA #103, from a 1 mM stock solution, was titrated into 100 μM [U- 15N] C2 dissolved in NMR buffer (10 mM sodium phosphate, pH 7.5, 100 mM Na2S2O3, 0.02% (w/v) NaN3, 90%/10%H2O/D2O) to a molar ratio of 2∶1. The titration was monitored by collecting 1H{15N}-HSQC spectra. (A) C2 domain NMR signal intensity changes were calculated by ΔI = (If – Ib)/If, where If(b) is the NMR signal intensity of free or PA #103-bound C2. Most of the C2 residues exhibited uniform broadening upon complex formation. Residues that exhibited signal broadening above 25% were considered to constitute the C2 interaction surface. (B) C2 domain chemical shift changes (Ω) were calculated by Ω = ((ΔδH)2 + (ΔδN/4)2)1/2, where ΔδH and ΔδN are the changes in amide proton and nitrogen chemical shifts, respectively. Residues that exhibited chemical shift changes above 0.01 ppm, were considered to constitute the C2 interaction surface. Cut-offs for selecting residues involved in the interaction between C2 and PA #103 are indicated by red arrows. The lower degree of signal broadening and chemical shift changes for the PA #103-C2 complex, as compared to PA #44-VC1, correspond to weaker binding (see Figure 3C). (DOCX) [file pone.0065180.s008.docx]

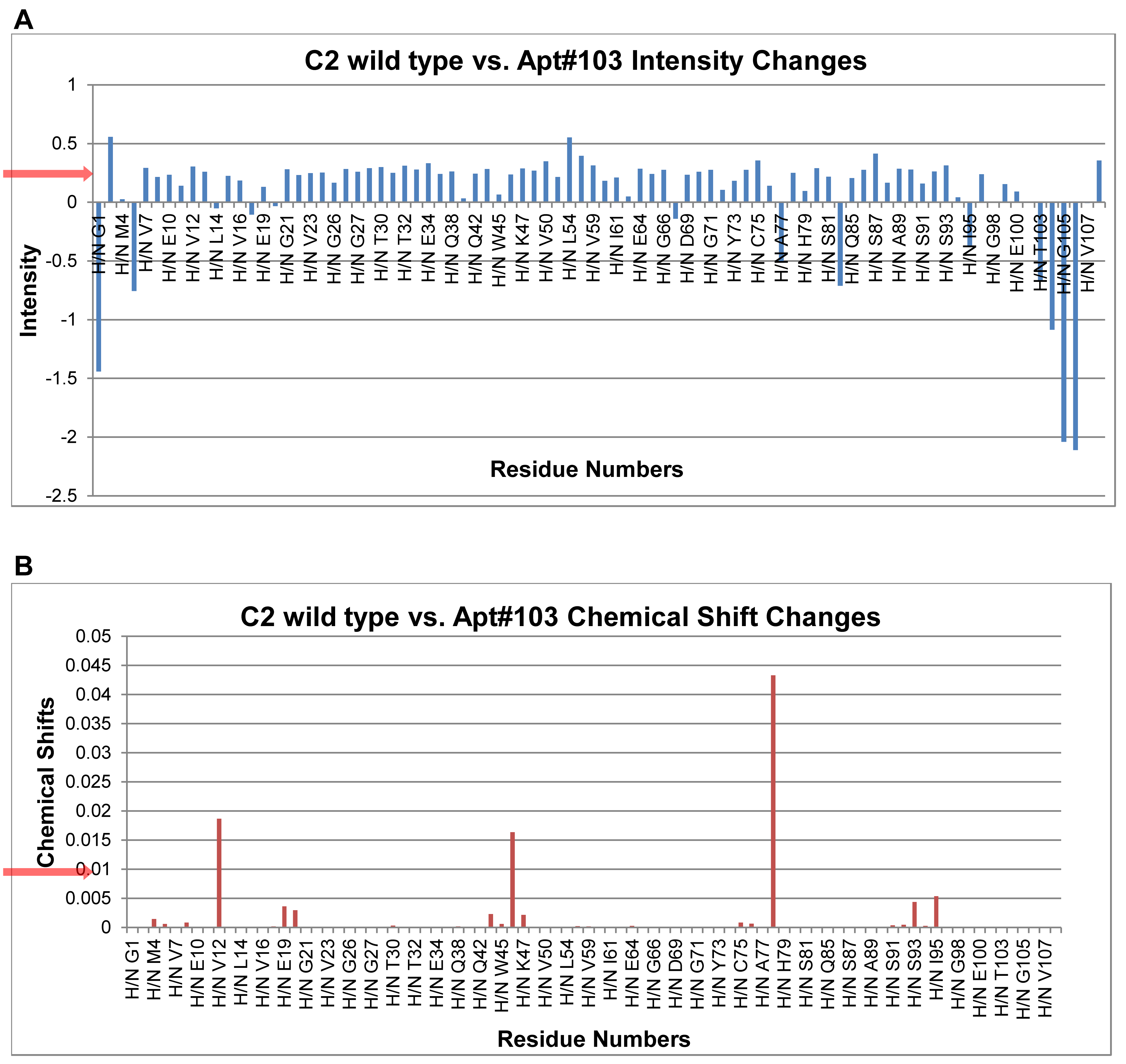


**Figure S8.** Changes in [*U-*^15^N] C2 NMR signal intensities and chemical shifts due to PA #103 binding. Unlabeled PA #103, from a 1 mM stock solution, was titrated into 100 μM [*U-*^15^N] C2 dissolved in NMR buffer (10 mM sodium phosphate, pH 7.5, 100 mM Na_2_S_2_O_3_, 0.02% (w/v) NaN_3_, 90%/10%H_2_O/D_2_O) to a molar ratio of 2:1. The titration was monitored by collecting ^1^H,^15^N-HSQC spectra. (**A**) C2 domain NMR signal intensity changes were calculated by

$$\Delta I=(I_{f}-I_{b})/I_{f}$$

where $I_{f(b)}$ is the NMR signal intensity of free or PA #103-bound C2. Most of the C2 residues exhibited uniform broadening upon complex formation. Residues that exhibited signal broadening above 25% were considered to constitute the C2 interaction surface. (**B**) C2 domain chemical shift changes (Ω) were calculated by

$$\Omega=\sqrt{\Delta\delta_{H}^{2}+(\Delta\delta_{N}/4)^{2}}$$

where $\Delta\delta_{H}$and $\Delta\delta_{N}$are the changes in amide proton and nitrogen chemical shifts, respectively. Residues that exhibited chemical shift changes above 0.01 ppm, were considered to constitute the C2 interaction surface. Cut-offs for selecting residues involved in the interaction between C2 and PA #103 are indicated by red arrows. The lower degree of signal broadening and chemical shift changes for the PA #103-C2 complex, as compared to PA #44-VC1, correspond to weaker binding (see Figure 3C).
