# Supplementary material for: Combinatorial Library of Improved Peptide Aptamers, CLIPs to Inhibit RAGE Signal Transduction in Mammalian Cells
Source: PLoS One. 2013 Jun 13;8(6):e65180. doi: 10.1371/journal.pone.0065180 (PMC3681763; doi:10.1371/journal.pone.0065180)
Supplement: Table S1 — List of oligonucleotides used. (DOCX) [file pone.0065180.s009.docx]

**Table S1. List of oligonucleotides used in this work**

|  | **Oligonucleotide sequence** | **Description** |
| --- | --- | --- |
| 1 | CGATTTCTGGGCAGAGTGGATCCTTGAATTCGGGCCCAAAATGATCGCCCCG | Introduction of PA insertion site in TrxA by mutagenesis |
| 2 | CGGGGCGATCATTTTGGGCCCGAATTCTAGGATCCACTCTGCCCAGAAATCG |  |
| 3 | TATGCTCTAGAC | Insertion of NdeI-(XbaI)-XhoI linker into pGADT7 |
| 4 | TCGAGTCTAGAGCA |  |
| 5 | TTTGGTACCGCCGCCATGGGCGATAAAATTATTCACCTGAC | Cloning of TrxA into pGADT7 at Kpn I site |
| 6 | AATGGTACCCAGGTTAGCGTCGAGGAACTCTTTC |  |
| 7 | GGCAGAGTGGATCCAANNKNNKNNKNNKNNKNNKNNKNNKAAGGGGCCCCTT | Random library oligonucleotide |
| 8 | AACTCCAAGCTTTGCAAAGATGGAT | Amplification of TrxA-Gal4 AD fragment from yeast colony |
| 9 | AGTGAACTTGCGGGGTTTTTCAGTATCTACGAT |  |
| 10 | TTTTTTCCATGGGCCATCATCATCATCATCATGATAAAATTATTCACCTGACTGACG | Recloning of TrxA-embedded PAs into pBAD plasmid at NcoI-HindIII sites |
| 11 | CCCAAGCTTACGCCAGGTTAGCGTCGAGGAACTC |  |
| 12 | TTTTATCGATGGGCTCAAAACATCACAGCCCGGATT | Cloning of RAGE VC1 into pRSET/EmGFP plasmid at ClaI-EcoRI sites |
| 13 | TTTTGAATTCACCTTCTGGCTCCACCACCAATTGG |  |
| 14 | TTTTATCGATGGGAGCCTGTGCCTCTGGAGGAGGT | Cloning of RAGE C2 into pRSET/EmGFP plasmid at ClaI-EcoRI sites |
| 15 | TTTTGAATTCTGATCCTCCCACAGAGCCTGCAG |  |
| 16 | GACGACAGTTTTGACACGAATGTACTCAAAGCGGACGG | Forward and reverse primers for D15N TrxA mutagenesis |
| 17 | CCGTCCGCTTTGAGTACATTCGTGTCAAAACTGTCGTC |  |
| 18 | CGGGGCGATCCTCGTCGCTTTCTGGGCAGAGTGGAT | Forward and reverse primers for D26A TrxA mutagenesis |
| 19 | ATCCACTCTGCCCAGAAAGCGACGAGGATCGCCCCG |  |
| 20 | GCAAACTGACCGTTGCAGAACTGAACATCGATCAAAACC | Forward and reverse primers for K57E TrxA mutagenesis |
| 21 | GGTTTTGATCGATGTTCAGTTCTGCAACGGTCAGTTTGC |  |
| 22 | GCAAACTGACCGTTGCACAACTGAACATCGATCAAAACC | Forward and reverse primers for K57Q TrxA mutagenesis |
| 23 | GGTTTTGATCGATGTTCAGTTGTGCAACGGTCAGTTTGC |  |
| 24 | CATCCGTGGTATCGCGACTCTGCTGCTGTTCA | Forward and reverse primers for P76A TrxA mutagenesis |
| 25 | TGAACAGCAGCAGAGTCGCGATACCACGGATG |  |
| 26 | TGCTGCTGTTCAAAAACGGTCGTGTGGCGGCAACCAAAGTG | Forward and reverse primers for E85R TrxA mutagenesis |
| 27 | CACTTTGGTTGCCGCCACACGACCGTTTTTGAACAGCAGCA |  |
