# Supplementary material for: Combinatorial Library of Improved Peptide Aptamers, CLIPs to Inhibit RAGE Signal Transduction in Mammalian Cells
Source: PLoS One. 2013 Jun 13;8(6):e65180. doi: 10.1371/journal.pone.0065180 (PMC3681763; doi:10.1371/journal.pone.0065180)
Supplement: Table S2 — Structural Statistics from the HADDOCK restrained calculations of the C2–PA #103 complex. (DOCX) [file pone.0065180.s010.docx]

**Table S2. Structural Statistics from the HADDOCK restrained calculations of the anti-C2 PA #103 complex**
