# Supplementary material for: Combinatorial Library of Improved Peptide Aptamers, CLIPs to Inhibit RAGE Signal Transduction in Mammalian Cells
Source: PLoS One. 2013 Jun 13;8(6):e65180. doi: 10.1371/journal.pone.0065180 (PMC3681763; doi:10.1371/journal.pone.0065180)
Supplement: Table S3 — HADDOCK AIRs restrains used to calculate C2-PA #103 complex. (DOCX) [file pone.0065180.s011.docx]

**Table S3. HADDOCK AIRs restrains used to calculate C2-PA #103 complex**

|  | Active Residues | Passive Residues |
| --- | --- | --- |
| RAGE C2 domain | Glu16, Glu18, Glu19, Ser92, Ile94, Glu95 | Val14, Val15, Pro17, Gly20, Ala21, Val22, Ala23, Ile91, Ile93, Pro96 |
| PA #103 | Arg41, Val42, Arg43, met44, Lys45, leu46, Val47, Lys48, Leu46, Val47, Lys48 | Trp37, Ile38, Gln39, Leu40, Gly49, Pro50, Lys51, Met52 |
